# The Public’s Awareness of and Attitude Toward Research Biobanks – A Regional German Survey

**DOI:** 10.3389/fgene.2018.00190

**Published:** 2018-05-24

**Authors:** Sabine Bossert, Hannes Kahrass, Daniel Strech

**Affiliations:** Institute for History, Ethics and Philosophy of Medicine, Hannover Medical School, Hannover, Germany

**Keywords:** public attitudes, postal survey, Germany, awareness of biobanks, biobank research

## Abstract

**Background:** Biobanks have become an increasingly important means of biomedical research and innovation. However, they entail a variety of ethical, social and legal challenges, which need to be publicly discussed and managed collectively. A certain level of public awareness of biobank research is an important prerequisite for the public to form an opinion on the issue at hand and to be willing to participate in public engagement activities. For many countries, including Germany, recent information on the public’s awareness of and attitude toward biobanks is scarce.

**Methods:** Therefore, by means of a postal survey in a German urban region, this study updates data from the 2010 Eurobarometer by analyzing (1) the public’s awareness of biobanks, (2) their general attitude toward biobanks, and (3) their hypothetical willingness to donate their own biological samples and personal or medical data.

**Results:** Overall, 204 (20.4%) of 998 delivered questionnaires were returned. The majority of survey respondents stated a positive attitude toward medical research (95.5%) and – to a somewhat lower degree – toward genetic research (61.3%). Attitudes toward biobanks were mixed but positive for the majority of respondents: in a question about their spontaneous assessment of biobanks as a means for medical research, 77% showed positive attitudes toward biobanks (36.6% “definitely” and 40.5% “somewhat positive”). This finding is also reflected in a high proportion of individuals willing to participate in biobank research: 70.4% of respondents would be willing to donate biomaterial to a biobank during a hypothetical stay in hospital. In spite of the high overall support respondents show for biobanks (e.g., positive general attitude and willingness to participate), only about one third (30.8%) had previously heard of biobanks.

**Discussion and Conclusion:** The comparison of survey results with prior data from the 2010 Eurobarometer indicates that public awareness of biobanks remains low. A higher level of biobank awareness can be assumed to be one prerequisite for public engagement in future decisions on biobank governance. We therefore argue that to increase public awareness of biobanks and to enable public involvement in biobank governance, publicly available and understandable information must be provided and disseminated.

## Introduction

In recent years an increasing number of biobanks in Europe and all over the world have been instituted, to collect and store human biological samples and related personal and medical data for indefinite periods of time. The samples and data stored in these biobanks can be used to address various issues in health and biomedical research, including basic research, questions in personalized or stratified medicine (involving e.g., genetic and other biomarkers), and research on widespread as well as rare diseases (Zika et al., 2010; Gottweis et al., 2012). Hence, biobanking is seen as an increasingly important means of biomedical research and innovation (Zika et al., 2010; Rahm et al., 2013). At the same time, biobank research entails a number of ethical, legal, and social issues (ELSI) as well as governance challenges (Meslin and Quaid, 2004; Petersen, 2005; Cambon-Thomsen et al., 2007; Hoeyer, 2008; Budin-Ljøsne et al., 2012; Hoeyer, 2012). These challenges include regulatory and oversight issues, recruitment of participants, data security, questions of returning research results, and informed consent (Budin-Ljøsne et al., 2012; Rial-Sebbag and Cambon-Thomsen, 2015). While these challenges are addressed mostly by multidisciplinary experts and governance actors, Gaskell and Gottweis point out that “biobanks need publicity” (Gaskell and Gottweis, 2011). At least two reasons for promoting public awareness and public discourse on biobank research can be identified in the literature: firstly, publicly discussing opportunities and challenges of biobank research is assumed to increase trust and thereby enhance the public’s support – e.g., willingness to participate and approval of public funding – for biobank research (McWhirter et al., 2014; Husedzinovic et al., 2015). Secondly, according to the Nuffield Council of Bioethics, for certain “emerging biotechnologies” public discourse is an ethical and practical requirement to address new governance and ELSI challenges (Nuffield Council on Bioethics, 2012). Since biobanks are clearly an emerging biotechnology, this discourse-ethical argument applies to this field as well (Dhai and Mahomed, 2013). A certain level of public awareness of biobank research is an important prerequisite for the public to form an opinion on the issue, and to be willing to participate in public engagement activities dealing with the ELSI and governance challenges they entail.

In response to the need for public awareness of and discourse about biobank research, there is a growing number of instances of the involvement of potential donors, patients, and the public at large. A review by Lander et al. (2014) reveals that the most popular area for public involvement activities in biomedical research and innovation is indeed biobanking. Many such activities merely assess participants’ views on certain issues by means of quantitative surveys or interviews. For example, some studies assess the public’s and (potential) tissue donors’ perspectives on informed consent (Hoeyer, 2010; D’Abramo et al., 2015; Ewing et al., 2015), privacy (Pulley et al., 2008; Kaufman et al., 2009), reporting of incidental findings (Murphy et al., 2008; Meulenkamp et al., 2010), and willingness to participate in biobank research (Critchley et al., 2010; Johnsson et al., 2010; Tupasela et al., 2010; Rahm et al., 2013; Porteri et al., 2014). In some discursive events, lay participants were invited to engage in more elaborate discussions of various ethical and social implications of biobanks (O’Doherty and Burgess, 2009; Secko et al., 2009; Lemke et al., 2010; Molster et al., 2011).

Meaningful debates on complex and normatively challenging issues – such as biobanking-related ELSI and governance issues – require a certain level of knowledge from participants. To start with, they must know that biobanks exist. Further, a deeper understanding of the procedures, safeguards, risks, and benefits of biobanks is a condition for substantial participation. However, the 2010 Eurobarometer indicates a low level of public knowledge of biobanks (TNS Opinion and Social, 2010; Gaskell et al., 2010, 2013; Budin-Ljøsne et al., 2012).

Considering this finding, Budin-Ljøsne et al. (2012) include the “lack of knowledge and lack of public debate surrounding biobank research” in their list of major ELSI challenges in biobanking. Because for many countries, including Germany, recent information on the public’s awareness of and attitude toward biobanks is scarce, in 2015 we conducted a postal survey in an urban region in Germany addressing the following objectives. Our first aim was to update findings from the 2010 Eurobarometer survey by collecting recent data on (1) the public’s awareness of biobanks, (2) their general attitude toward biobanks, and (3) their hypothetical willingness to donate their own biological samples and personal or medical data. Our second aim was to place the updated survey results in the context of respondents’ socio-demographics and experiences with and attitude toward medical research.

The postal survey was part of a larger project aimed at user-testing and revising an informed consent form for biobank research (Bossert et al., 2017).

## Materials and Methods

### Postal Survey

In spring 2015 we conducted a postal survey which was sent to 1,050 inhabitants of the region of Hannover. The sample was randomly selected by the Hannover registration office from the register of all residents (age 18 or older). Three weeks after the initial mailing all addressees who had not yet returned the questionnaire or actively withdrawn from the study (e.g., by telephone call) were sent a personalized reminder containing the whole survey package (cover letter, study information, informed consent form and questionnaire). No payment or other incentive was offered to survey participants. To allow for the analysis of response rates of different sub-groups, in addition to names and addresses the registration office also provided information about addressees’ gender and age. The questionnaire, designed by members of our research group and pre-tested in qualitative interviews with five contacts of two project assistants, contains 20 items, of which five are reproduced from the 2010 Eurobarometer questionnaire (awareness of biobanks and willingness to donate biological samples and personal or medical data) (Europäische Kommission, 2010; Gaskell et al., 2010). A list of all items used in the survey instrument is presented in the **Supplementary Text S1**. As in the Eurobarometer survey, the concept of biobanks is briefly introduced in our questionnaire: “In the next section, we are going to ask you some question about ‘biobanks.’ Biobanks collect human biomaterials (e.g., blood, urine, or tissue samples), link them with selected personal and medical data (e.g., age, gender, blood values, clinical history) and store them for an indefinite period of time. The biological material and clinical data are used in research to help improve the prevention, diagnosis and treatment of diseases.”

The other questionnaire items assess (1) respondents’ general attitude toward biobanks (1 item); (2) their attitude toward and experience with medical and genetic research (7 items), and (3) socio-demographic characteristics (7 items). To measure respondents’ assessment of biobanks, medical and genetic research, and their willingness to participate in biobank research, we presented four-point scales (e.g., “How do you assess biobanks as a means for medical research?” – “definitely positive – somewhat positive – somewhat negative – definitely negative”) and five-point scales (e.g., “Every form of research entails risks and benefits. Thinking of medical research, what do you believe?” – “Benefits clearly outweigh risks – Benefits somewhat outweigh risks – Benefits equal risks – Risks somewhat outweigh benefits – Risks clearly outweigh benefits.”). The project was approved by Hannover Medical School’s local research ethics committee (No. 6689-2014).

### Data Analysis

The main goal of data analysis was to describe respondents’ answers regarding the above-mentioned items and to explore possible influencing variables for variation in their responses. Hence, data analysis was mainly confined to frequency analyses and bivariate context analyses (especially χ^2^ tests). In addition, we also conducted binary logistic regression analyses for the variables “prior awareness of biobanks,” “spontaneous assessment of biobanks,” and “hypothetical willingness to donate biomaterial and data.” Results of the regression models are presented as **Supplementary SPSS Output S1**. Survey data were processed and analyzed using SPSS. Raw data are presented as **Supplementary Data Sheet S1**. Potential influencing factors included gender, age, and school education, as well as prior personal experience with medical research, prior working experience in the field of health or health care, and respondents’ general assessment of genetic research (a well-known field of biobank research). For the analysis of potential influencing variables, the age variable was divided into groups of 10 years (“29 years or younger,” “30–39”, …, “70–79”, “80 years or older”). School education was measured in six categories in the questionnaire (from “left school without qualifications” to “Abitur/A levels (highest German school qualification, requiring 12–13 years of education”). Two additional categories – “still going to school” and “other qualifications” were included. For analysis, the six categories were combined into three groups: “lower,” “middle,” and “higher level of school education.”

## Results

### Response Analysis and Characteristics of Survey Participants

Of the 1,050 survey addressees, 52 had either moved or died before the mailing, and hence were not available. 204 (20.4%) of the 998 available individuals participated in the survey. 122 (12.2%) individuals actively refused to participate in the survey, e.g., by telephone call, or by sending back a blank questionnaire.

The characteristics of survey respondents are shown in **Table 1**. In comparison with the data from the Hannover residency office, the group of survey respondents shows the following biases (see also **Supplementary Tables S1**, **S2**): men, members of younger age-groups, and persons with other than German nationality were underrepresented. Compared to data given in the German statistical yearbook (Federal Bureau of Statistics, 2015), individuals with lower and middle school education were also underrepresented in our study.

**Table 1:** Characteristics of survey respondents.

		Survey respondents	
Item		(*n*)	(valid %)^1^
Gender	Male	84	(41.6)
	Female	118	(58.4)
Age	29 or younger	32	(15.9)
	30–39 years	19	(9.5)
	40–49 years	34	(16.9)
	50–59 years	46	(22.9)
	60–69 years	23	(11.4)
	70–79 years	34	(16.9)
	80 years or older	13	(6.5)
	Mean; range	Mean: 53.0; quartiles: 37, 52, 67 min: 19; max: 101	
School education	Low	28	(13.9)
	Middle	47	(23.4)
	High	116	(57.7)
	“Other”	10	(5.0)
Ever worked in health care-sector?	Yes	159	(20.5)
	No	41	(79.5)
Prior experience in research (medicine or other)?	Yes	31	(15.4)
	No	170	(84.6)
Nationality	Solely German	187	(92.1)
	German plus “other“/solely “other”	16	(7.9)

*^1^Share of missing values between 0.0 and 4.4% of N = 204 respondents.*

### Attitude Toward Medical Research and Genetic Research

The vast majority of survey respondents stated that they definitely/somewhat approved of medical research (*n* = 194 of 204 respondents, **Table 2**) and most of them (*n* = 160) thought its benefits are greater than its risks. Due to the low variation in the assessment of medical research, this item was not included as a potential influencing factor for participants’ awareness of and attitude toward biobanks. Approval of genetic research was less definite. Not only did *n* = 34 individuals (somewhat or definitely) disapprove of genetic research in general, but almost one fifth (*n* = 40) of respondents were “not sure” how to assess this kind of research (**Table 2**). Reservations about genetic research also appeared in the assessment of its risks and benefits: *n* = 95 respondents thought the benefits of genetic research outweigh its risks.

**Table 2:** Assessment of medical and genetic research (*N* = 204).

	Medical research		Genetic research	
	*n*	(%)	*n*	(%)
**General attitude of medical and genetic research**				
1 “definitely approve”	146	(71.6)	45	(22.1)
2 “somewhat approve”	48	(23.5)	80	(39.2)
3 “somewhat disapprove”	1	(0.5)	26	(12.7)
4 “definitely disapprove”	0	(0.0)	8	(3.9)
Not sure	4	(2.0)	40	(19.6)
Not stated	5	(2.5)	5	(2.5)
Mean	1.26^∗^		1.98^∗^	
**Assessment of benefits and risks**				
1 “benefits clearly outweigh risks”	79	(34.3)	29	(14.2)
2 “benefits somewhat outweigh risks”	81	(39.7)	66	(32.4)
3 “benefits equate to risks”	1	(21.6)	26	(29.4)
4 “risks somewhat outweigh benefits”	2	(0.5)	13	(12.7)
5 “risks clearly outweigh benefits”	44	(1.0)	60	(6.4)
not stated	6	(2.9)	10	(4.9)
Mean	1.89^∗∗^		2.64^∗∗^	

*^∗^Differences in assessment of medical and genetic research are statistically significant: paired sample t-test: mean-difference = -0.72; t = -11.817; p = 0.000. ^∗∗^Differences in assessment of benefits and risks are statistically significant: paired sample t-test: mean-difference = -7.5; t = -10.630; p = 0.000.*

### Awareness of and Experiences With Biobank Research

About one third of respondents had already heard of biobanks (**Table 3**). The degree of knowledge varied significantly with respondents’ school education. No other variation shown in **Table 3** is statistically significant – including respondents’ prior working experience in medicine or health-care. Of the 62 respondents who had heard of biobanks before, 44 (71%) had at least once talked about biobanks with someone (**Table 4**). Only 20 (32.3%) had searched for information about biobanks and 8 (12.9%) had already donated biomaterial to a biobank.

**Table 3:** “Have you ever heard anything of biobanks before today?”

		Yes	No	Total (*n* = 100%)
**Numbers are valid %, proportion of missing values between 1.5 and 7.4%^8^**				
All^1^		30.8	69.2	(201)
Assessment of genetic research^2^	Somewhat/definitely approve	32.8	67.2	(125)
	Somewhat/definitely disapprove	26.5	73.5	(34)
	Not sure	30.0	70.0	(40)
Age^3^	29 or younger	25.0	75.0	(32)
	30–39 years	33.3	66.7	(18)
	40–49 years	26.5	73.5	(34)
	50–59 years	39.1	60.9	(46)
	60–69 years	39.1	60.9	(23)
	70–79 years	27.3	72.7	(33)
	80 years and older	15.4	84.6	(13)
Gender^4^	Male	32.5	67.5	(83)
	Female	29.1	70.9	(117)
School education^5,8,∗^	Low	3.6	96.4	(28)
	Middle	27.7	72.3	(47)
	High	37.7	62.3	(114)
Ever worked in health-care^6^	No, never	29.9	70.1	(157)
	Yes	34.1	65.9	(41)
Prior experience in research^7^	No	28.4	71.6	(169)
	Yes	45.2	54.8	(31)
Nationality^1^	Solely German	30.3	69.7	(185)
	German plus “other” / solely “other”	37.5	62.5	(16)

^1^1.5% missing values; ^2^2.5% missing values; ^3^2.5% missing values; ^4^2.0% missing values; ^5^7.4% missing values; ^6^2.9% missing values; ^7^2.0% missing values; ^8^high percentage of missing values for education due to exclusion of n = 10 “other kind of degree.” ^*^Differences statistically significant: χ^2^ = 12.630, p = 0.002.

**Table 4:** Experiences with biobanks (*N* = 62 respondents who already had heard of biobanks).

If you have heard of biobanks: have you ever…	Yes, frequently		Yes, occasionally		Yes, only once or twice		No, never		Not stated	
*N* = 62 respondents who already had heard of biobanks	*n*	(%)	*n*	(%)	*n*	(%)	*n*	(%)	*n*	(%)
… talked about biobanks with anyone?	1	(1.6)	18	(29.0)	25	(40.3)	17	(27.4)	1	(1.6)
… searched for information about biobanks?	1	(1.6)	6	(9.7)	13	(21.0)	37	(59.7)	5	(8.1)
… donated biomaterial yourself?	1	(1.6)	1	(1.6)	6	(9.7)	49	(79.0)	5	(8.1)

### Attitudes Toward Biobank Research

In a question about their spontaneous assessment of biobanks as a means for medical research, respondents showed mainly positive attitudes toward biobanks (36.6% “definitely” and 40.5% “somewhat positive”; see **Table 5**). The only significant independent variables explaining variation of respondents’ assessment of biobanks are their assessment of genetic research, and gender: male respondents and persons who somewhat or definitely approve of genetic research were more likely to approve of biobanks. These groups also show relatively low rates of “unsure” or absent responses.

**Table 5:** Spontaneous assessment of biobanks as a means for biomedical research.

		Definitely positive	Somewhat positive	Somewhat negative	Definitely negative	Not sure	Not stated	Total (*n* = 100%)
**Numbers are valid %, proportion of missing values between 1.0 and 6.7%^9^**							
All		36.6	40.5	3.4	1.5	13.7	4.4	(204)
Heard of biobanks before^1^	Yes	45.2	40.3	6.5	1.6	4.8	1.6	(62)
	No	33.1	41.7	2.2	1.4	17.3	4.3	(139)
Assessment of genetic research^2,^^∗^	Somewhat/definitely approve	45.6	40.0	3.2	0.0	8.8	2.4	(125)
	Somewhat/definitely disapprove	14.7	35.3	5.9	8.8	32.4	2.9	(34)
	Not sure	30.0	52.5	2.5	0.0	10.0	5.0	(49)
Age (quartiles)^3^	29 or younger	53.1	31.3	0.0	0.0	15.6	0.0	(32)
	30–39 years	26.3	47.4	5.3	0.0	15.8	5.3	(19)
	40–49 years	35.3	32.4	2.9	2.9	23.5	2.9	(34)
	50–59 years	28.3	58.7	4.3	2.2	4.3	2.2	(46)
	60–69 years	34.8	26.1	8.7	4.3	17.4	8.7	(23)
	70–79 years	44.1	35.3	0.0	0.0	14.7	5.9	(34)
	80 years and older	23.1	61.5	7.7	0.0	0.0	7.7	(13)
Gender^4,^^∗∗^	Male	42.9	41.7	2.4	3.6	7.1	2.4	(84)
	Female	31.4	40.7	4.2	0.0	18.6	5.1	(118)
School education^5,9^	Low	28.6	39.3	0.0	0.0	17.9	14.3	(28)
	Middle	34.0	48.9	2.1	0.0	12.8	2.1	(47)
	High	37.9	38.8	5.2	1.7	13.8	2.6	(116)
Ever worked in health-care^6^	No, never	34.0	44.7	2.5	1.9	13.2	3.8	(159)
	yes	48.8	29.3	7.3	0.0	14.6	0.0	(39)
Prior experience in research^7^	No	36.5	41.2	2.9	1.8	14.7	2.9	(170)
	Yes	38.7	41.9	6.5	0.0	9.7	3.2	(31)
Nationality^8^	Solely German	36.4	41.2	3.7	0.5	14.4	3.7	(187)
	German plus “other” /solely “other”	37.5	37.5	0.0	12.5	6.3	6.3	(16)

^1^1.5% missing values; ^2^2.5% missing values; ^3^1.5% missing values; ^4^1.0% missing values; ^5^9.8% missing values; ^6^2.0% missing values; ^7^1.5% missing values; ^8^0.5% missing values; ^9^high percentage of missing values for education due to exclusion of n = 10 “other kind of degree.” ^*^Differences statistically significant: (χ^2^ = 36.738, p = 0.000); ^**^Differences statistically significant: (χ^2^ = 12.098, p = 0.033).

Irrespective of the mainly positive assessment of biobanks, 13.7% were not sure about their assessment and 4.4% did not answer this question. **Table 6** shows that respondents who claimed prior awareness of biobanks, made a positive assessment of genetic research, or identified as male, were significantly more likely to make a definite assessment of biobanks (i.e., giving clear “yes” or “no” statements).

**Table 6:** Spontaneous assessment of biobanks: predictors of definite answer (clear “yes” or “no” statements, not counting missing values and “unsure” statements).

		Assessment of biobanks		
		Definite answer	No definite answer (“not sure” or no answer)	Total (*n* = 100%)
**Numbers are valid %, proportion of missing values between 0.5% and 6.4%**				
All		81.9	18.1	(204)
Heard of biobanks before^1,^^∗^	Yes	93.5	6.5	(62)
	No	78.4	21.6	(139)
Assessment of genetic research^2,^^∗∗^	Somewhat/definitely approve	88.8	11.2	(125)
	Somewhat/definitely disapprove	64.7	35.3	(34)
	Not sure	85.0	15.0	(40)
Age (quartiles)^3^	29 or younger	84.4	15.6	(32)
	30–39 years	78.9	21.1	(19)
	40–49 years	73.5	26.5	(34)
	50–59 years	93.5	6.5	(46)
	60–69 years	73.9	26.1	(23)
	70–79 years	79.4	20.6	(34)
	80 years and older	92.3	7.7	(13)
Gender^4,^^∗∗∗^	Male	90.5	9.5	(84)
	Female	76.3	23.7	(118)
School education^5,9^	Low	67.9	32.1	(28)
	Middle	85.1	14.9	(47)
	High	83.6	16.4	(116)
Ever worked in health-care^6^	No, never	83.0	17.0	(159)
	Yes	85.4	14.6	(41)
Prior experience in research^7^	No	82.4	17.6	(170)
	Yes	87.1	12.9	(31)
Nationality^8^	Solely German	81.8	18.2	(187)
	German plus “other” / solely “other”	87.5	12.5	(16)

^1^1.5% missing values; ^2^2.5% missing values; ^3^1.5% missing values; ^4^1.0% missing values; ^5^6.4% missing values; ^6^2.0% missing values; ^7^1.5% missing values; ^8^0.5% missing values; ^9^high percentage of missing values for education due to exclusion of n = 10 “other kind of degree.” ^*^Differences statistically significant: (χ^2^ = 36.985, p = 0.008); ^**^Differences statistically significant: (χ^2^ = 11.542, p = 0.033); ^***^Differences statistically significant: (χ^2^ = 6.761, p = 0.009).

### Willingness to Participate in Biobank Research

As **Table 7** shows, a majority of survey respondents (70.4%) would be willing to donate biomaterial to a biobank during a hypothetical stay in hospital: 28.6% chose the answer “yes, certainly,” and 41.8% chose “yes, probably.” Unsurprisingly, a positive assessment of genetic research and biobanks, as well as prior working experience in health care, increased the probability of a respondent being willing to participate in biobank research (**Table 7**). However, knowledge of biobanks, gender, and school education show no significant influence.

**Table 7:** Willingness to donate biomaterial (hypothetical setting).

		Yes, certainly	Yes, probably	No, probably not	No, certainly not	Not sure	Total (*n* = 100%)
**Numbers are valid %, proportion of missing values between 3.9 and 9.8%^11^**						
All^1^		28.6	41.8	11.7	7.1	10.7	(196)
Assessment of genetic research^2,^^∗^	Somewhat/definitely approve	33.6	46.7	9.0	4.1	6.6	(122)
	Somewhat/definitely disapprove	5.9	35.3	20.6	20.6	17.6	(34)
	Not sure	34.2	34.2	7.9	5.3	18.4	(38)
Heard of biobanks before^3^	Yes	34.4	37.7	11.5	6.6	9.8	(61)
	No	26.1	44.0	11.2	7.5	11.2	(134)
Spontaneous assessment of biobanks^4,^^∗∗^	Definitely/somewhat positive	34.8	47.1	7.7	1.9	8.4	(155)
	Definitely/somewhat negative	0	0	40.0	50.0	10.0	(10)
	Not sure	3.8	23.1	26.9	19.2	26.9	(26)
Age^5^	29 or younger	38.7	35.5	12.9	3.2	9.7	(31)
	30–39 years	16.7	50.0	22.2	5.6	5.6	(18)
	40–49 years	31.3	37.5	6.3	6.3	18.8	(32)
	50–59years	23.9	52.2	6.5	6.5	10.9	(46)
	60–69 years	21.7	34.8	17.4	17.4	8.7	(23)
	70–79 years	36.4	45.5	6.1	6.1	6.1	(33)
	80 years and older	25.0	25.0	25.0	8.3	16.7	(12)
Gender^6^	Male	29.6	48.1	8.6	4.9	8.6	(81)
	Female	27.8	37.4	13.9	8.7	12.2	(115)
School education^7,11^	Low	15.4	46.2	7.7	7.7	23.1	(26)
	Middle	26.1	52.2	8.7	4.3	8.7	(46)
	High	32.1	36.6	13.4	8	9.8	(112)
Ever worked in health-care^8,^^∗∗∗^	No, never	25.5	45.2	7.7	8.4	13.5	(155)
	Yes	41.0	28.2	28.2	2.6	0.0	(39)
Prior experience in research^9^	Yes	26.5	42.8	11.4	7.2	12.0	(166)
	No	40.0	36.7	13.3	6.7	3.3	(30)
Nationality^10^	Solely German	29.4	42.8	11.7	6.1	10.0	(180)
	German plus “other” / solely “other”	18.8	31.3	12.5	18.8	18.8	(16)

^1^3.9% missing values; ^2^4.9% missing values; ^3^4.4% missing values; ^4^6.4% missing values; ^5^4.4% missing values; ^6^3.9% missing values; ^7^9.8% missing values; ^8^4.9% missing values; ^9^3.9% missing values; ^10^3.9% missing values; ^11^high percentage of missing values for education due to exclusion of n = 10 “other kind of degree.” ^*^Differences statistically significant: (χ^2^ = 28.568, p = 0.000); ^**^Differences statistically significant: (χ^2^ = 77.267, p = 0.000); ^***^Differences statistically significant: (χ^2^ = 22.765, p = 0.000).

## Discussion

### Assessment of Medical and Genetic Research

In our survey 95.1% of all respondents definitely/somewhat approved of medical research and 61.3% definitely/somewhat approved of genetic research. The high levels of support for medical research and for genetic research confirm the results of earlier studies. For example, in a survey of the public’s perceptions of biomedical research in Ireland (Cousins et al., 2005), 96% of the respondents agreed with the statement “medical research is important because it results in new and improved treatments for diseases.” 87% agreed that “medical research helps us live longer” (ibid.). As we also found, in the Irish survey support for genetic research is still high but somewhat lower than for medical research: 73% agreed with the statement “New genetic developments will bring cures for many diseases” (ibid.). However, 42% agreed that “research on human genetics is tampering with nature,” which shows respondents’ reservations regarding this kind of research. Similarly to our sample, the proportion of those who were “unsure” how to assess the given statements was higher for genetic research than for medical research (ibid.). The relatively high proportion of 19.6% “unsure” answers in our survey is also reflected in a survey amongst hospital patients in six hospitals in the State of New York (United States) (Kerath et al., 2013). In this survey, 16.9% of respondents were unsure how they “feel about genetic research.” However, almost all other participants (82.2%) unequivocally approved of genetic research (only 0.9% disapproval).

### Comparison of Results With Eurobarometer Results From 2010

Like the 2010 Eurobarometer, we conducted a postal survey of the public’s awareness of and attitude toward biobanks, as well as their willingness to donate biological samples and personal or medical data. Our survey repeated 5 items from the 2010 Eurobarometer (TNS Opinion and Social, 2010). However, while the Eurobarometer surveyed European citizens from 27 countries, we recruited our sample population solely from the registration office in Hannover (an urban region with about 1.1 million inhabitants). Also, different survey methods were used: standardized personal interviews for the Eurobarometer, postal questionnaires in our study. Alongside different response rates, the proportions of male and female respondents differ between the samples: 48.3% males in Eurobarometer, 41.6% in our survey. However, the response rates of different age groups and of individuals with different levels of education are comparable.

### Prior Awareness of Biobanks

One important result of the comparison between the surveys concerns respondents’ awareness of biobanks: although biobanks are an increasingly important means of biomedical research and the number of biobanks in Germany is increasing, the degree of the public’s knowledge of biobanks remains at a rather low level. In the Eurobarometer survey, 34% of all Europeans and 29% of German respondents stated that they had heard of biobanks before (Europäische Kommission, 2010; TNS Opinion and Social, 2010; Gaskell et al., 2013). In our survey (about 5 years later) 30.8% of respondents chose this answer. Even compared with four other biotechnological innovations which were included in the Eurobarometer survey, awareness of biobanks may be considered low [compared with genetically modified foods (95% awareness by German respondents), animal cloning in food production (87%), nanotechnology (65%), and synthetic biology (18%)] (Gaskell et al., 2010). Therefore, the conclusion of Gaskell and colleagues from the Eurobarometer survey that “biobanks have not done enough to generate engagement among the public” (Gaskell et al., 2013) still seems to apply.

Also, in both surveys, individuals with higher levels of education were more likely to have already heard of biobanks. This difference is notable in two regards: firstly, awareness in the general public could be even lower than in the survey samples, because individuals with a higher level of education were over-represented in both surveys. Secondly, if biobank awareness is to be raised, measures to reach less highly educated persons should be taken. These could include easy-to-read information materials, public lectures, and broader media coverage.

In contrast with the Eurobarometer results, in our survey most respondents who had heard of biobanks before had talked to someone about biobanks at least once (70.9% compared to 49% of German and 48% of all respondents in the Eurobarometer data). Also, the proportion of those who had actively searched for information about biobanks was higher in our survey (32.3% compared to 22% of German and 24% of all respondents in Eurobarometer). These differences may hint at increased public interest in biobanks: at least those who have heard of biobanks seem to be more interested in talking about them or learning more about them.

### Hypothetical Willingness to Participate and General Assessment of Biobanks

The tendency toward a more open attitude regarding biobanks in our data is also reflected in a high proportion of respondents who expressed willingness to donate biomaterial to a biobank (70.4% compared to 42% of German and 46% of all respondents in the Eurobarometer data). However, the higher willingness compared to the Eurobarometer survey could be – at least partly – explained by different wordings of the items in the survey instruments. While both surveys used hypothetical scenarios to assess respondents’ willingness to participate in biobank research, the questions varied in their level of detail: “Would you be willing to provide information about yourself to a biobank?” in the Eurobarometer vs. “Would you be willing to donate tissue or blood samples to a biobank, if those materials would be left over after a medical treatment in hospital?” in our survey. Other surveys – e.g., in the United States (69%) (Rahm et al., 2013), Finland (83%) (Tupasela et al., 2010), and Italy (86%) (Porteri et al., 2014) – match the high rates of willingness in our survey. However, a review of hypothetical and actual willingness to participate in biobank research in six countries shows a broad range of willingness in hypothetical scenarios (40–96%) as well as actual participation rates (10–98%) (Johnsson et al., 2010). This indicates that results on general support for biobanks have to be carefully interpreted, because they seem to vary considerably between populations, settings and survey methods. The high levels of support for biobanks in our survey could also be a result of self-selection processes during the survey (see also Methodological Restrictions) – e.g., if persons with a positive attitude toward research in general were more likely to participate in our survey, they can be assumed to be more supportive toward biobanks, too.

While in other studies, including the Eurobarometer survey, willingness to participate in biobank research increased with years of education, in our study willingness was highest for individuals in the category of “middle education” (Tupasela et al., 2010; Gaskell et al., 2013; Porteri et al., 2014). In our survey another strong predictor for respondents’ willingness to participate was a positive attitude toward genetic research (80.3% vs. 41.2% willingness rates). In the Eurobarometer data, willingness to participate was higher for respondents who had heard of biobanks before (62% compared to 38%). Although this result was not confirmed by our data (72.1% vs. 70.1% willingness to participate for persons with and without prior knowledge of biobanks), respondents with prior knowledge of biobanks in our survey were significantly more likely to give a definite assessment of biobanks (6.4% vs. 21.6% “unsure” or no response). Also, for willingness to participate and for spontaneous assessment of biobanks, persons who had heard of biobanks before seemed to be more certain of their response. This is indicated by the higher proportions of “yes, certainly” and “definitely positive” compared to “probably yes” and “somewhat positive” for those respondents.

In sum, based on both surveys, it can be assumed that individuals are either more open to subjects they already know about (Eurobarometer) or are more certain (our survey) about their answers when they have at least an idea of what biobanks are.

### Methodological Restrictions

The survey presented here was part of a larger research project whose aim was to engage members of the public in focus-group discussions to optimize informed consent for biobank research. Because of the scope of this project, our survey only addressed residents of the German city of Hannover. Moreover, the response rate after one personalized postal reminder was 20.4% (*N* = 204). This rather low response rate might have caused biases in the survey results. Therefore, the generalisability of our survey’s results to the whole German population may be limited. However, a low response rate is a very common problem for survey-based research, especially when addressees are confronted with abstract and normatively challenging questions (Edwards et al., 2002). Our survey included several items on complex issues which were new to many respondents (see relatively high numbers of “not sure” or absent responses); this could be one explanation for the low response rates. In general, lower response rates are reported when participants are sampled from the public arena rather than a clinical or research setting (Cousins et al., 2005). For example, surveys in the United States (Schwartz et al., 2001) and Sweden (Ring and Lindblad, 2003) achieved respective response rates of 20 and 30% in their studies of informed consent in biobanking. A telephone survey of the public’s willingness to participate in biobank research showed similarly low response rates (Critchley et al., 2010). Some other surveys on similar issues used samples of patients and their family members or actual study participants (Pulley et al., 2008; Hoeyer, 2010; Rahm et al., 2013; Porteri et al., 2014). These studies usually do not provide response analyses because they do not know the characteristics of their basic population. We aimed to survey the perspectives of the general public, and therefore used a representative sample of all inhabitants of the Hannover region. This enabled us to at least show biases in age, gender, and nationality of our study population in comparison with the original sample. The biases shown by our study population are consistent with those of a Finnish survey on similar issues (Tupasela et al., 2010).

However, in spite of the limitations regarding the base population and response rate, the above-mentioned similarities to some results of the 2010 Eurobarometer indicate that data from our regional survey are in principle comparable to data from a survey conducted at a national level. Furthermore, due to the lack of recent data for Germany, our survey provides a first update of the public’s – rather than patients’ or prior study-participants’ – knowledge of and attitude toward biobank research and, thus, can help to further explore this field.

## Conclusion

It has been argued that with the increasing importance for biomedical research of biobanks, and the governance challenges they face (Gottweis et al., 2012), it is important to engage the public when addressing these challenges (Gaskell and Gottweis, 2011). As has already been shown in other fields of research and governance, qualitative methods or deliberative formats are most suitable for meaningful and effective involvement of the public in governance decision-making (Abelson et al., 2003, 2004; Secko et al., 2009; Niemeyer, 2011). Surveys like ours, however, can be used to explore the field and to identify challenges for more advanced methods of public participation.

Our survey shows, for example, that in spite of the increasing importance of biobanks for biomedical research, and although their number has been increasing in recent years, the public’s awareness of biobanks in Germany remains low. However, in comparison with prior survey results, our data indicate that individuals who had heard of biobanks before were more likely to engage in discussions with others and to seek further information. Hence, once they are aware of it, individuals seem to be interested in engaging with the topic. Also, in our survey individuals with prior awareness of biobanks were significantly more likely to state a definite attitude toward biobanks (higher percentage of persons making a clear “yes” or “no” statement, lower percentage of “not sure” and missing statements). Thus, among other variables – e.g., gender and attitude toward genetic research – awareness of biobanks seems to be one predictor for the formation of an opinion toward this issue. This conclusion is also supported by experiences with prior deliberative events on biobanking (O’Doherty and Burgess, 2009; Secko et al., 2009; Molster et al., 2011; O’Doherty et al., 2012), which indicate that meaningful public engagement with biobank issues primarily depends on people learning about biobanks and being given the opportunity to form their own opinions of the corresponding governance challenges. This requires an open and unbiased dialog between biobank experts and members of the public – e.g., by means of organized and planned deliberative procedures or by informal events such as open days, popular lectures and discussions, and behind-the-scenes tours.

The aim of our study was not to assess the accuracy of the public’s knowledge of biobanks, but their general awareness that biobanks existed and their spontaneous attitude toward biobanks. However, future studies addressing the public’s actual knowledge – e.g., facts, misunderstandings, and information gaps about biobanks – could help to develop publicly available and easily understandable information materials about biobanks, which would meet the public’s information needs more effectively. Only on the basis of comprehensive information can members of the public form reliable and stable opinions on biobanks, which is a prerequisite for meaningful public participation in these issues. The efforts made in this regard should be evaluated and if necessary refined to encourage and truly enable the public to engage in discussions on ELSI and governance issues regarding biobank research.

## Ethics Statement

The paper presents data from a postal Survey. Each survey participant was offered the option to participate in the study by voluntarily filling in and sending back the questionnaire; or not to participate in the study and throw away the questionnaire or send back a blank questionnare. No vulnerable populations were involved in the study. Hannover Medical School’s Research ethics committee has approved the study in Spring 2015 (No. 6689-2014) after reviewing all study materials and the study protocol.

## Author Contributions

SB, DS, and HK conceived and designed the study and acquired the data. SB analyzed the data and drafted the manuscript. HK and DS substantially contributed to data analysis and critically revised the manuscript. All authors approved the final version of the manuscript and agreed to be accountable for all aspects of the work.

## Conflict of Interest Statement

The authors declare that the research was conducted in the absence of any commercial or financial relationships that could be construed as a potential conflict of interest.

## Supplementary Material

TABLE S1 Response rate by age and gender.

TABLE S2 Response rate by nationality.

TEXT S1 List of questions included in the questionnaire.

DATA SHEET S1 Raw Data used for analysis.

SPSS OUTPUT S1 Binary logistic regression analyses.
