# Supplementary material for: The Public’s Awareness of and Attitude Toward Research Biobanks – A Regional German Survey
Source: Front Genet. 2018 May 24;9:190. doi: 10.3389/fgene.2018.00190 (PMC5977155; doi:10.3389/fgene.2018.00190)
Supplement: TABLE S1 — Response rate by age and gender. [file Table_1.docx]

Supplementary table S1: response rate (survey) by age and gender

| **age groups** | **gender** | **n (persons available)** | **response rate** | | **response rate** | |
| --- | --- | --- | --- | --- | --- | --- |
|  |  |  | **% of male/female for each age group** | **(n)** | **% of age group (both genders)** | **(n)** |
| **over all age groups** | **male** | 512 | 17.2 | (87) | 20.4 | (204) |
|  | **female** | 485 | 23.9 | (117) |  |  |
| **18-29** | **male** | 97 | 13.4 | (13) | 16.7 | (31) |
|  | **female** | 89 | 20.2 | (18) |  |  |
| **30-39** | **male** | 98 | 9.2 | (9) | 13.0 | (22) |
|  | **female** | 71 | 18.3 | (13) |  |  |
| **40-49** | **male** | 90 | 16.7 | (15) | 21.1 | (35) |
|  | **female** | 76 | 26.3 | (20) |  |  |
| **50-59** | **male** | 92 | 26.1 | (24) | 27.4 | (45) |
|  | **female** | 72 | 29.2 | (21) |  |  |
| **60-69** | **male** | 59 | 10.2 | (6) | 20.5 | (24) |
|  | **female** | 58 | 31.0 | (18) |  |  |
| **70-79** | **male** | 57 | 26.3 | (15) | 27.2 | (34) |
|  | **female** | 68 | 27.9 | (19) |  |  |
| **≥ 80** | **male** | 19 | 31.6 | (6) | 18.6 | (13) |
|  | **female** | 51 | 13.7 | (7) |  |  |
| Differences in response rates tested by means of chi^2^-test; χ^2^= 18,667, p=0.005 (two-sided) N=997 (for one individual there was no age given in Hannover residency data or questionnaire) | | | | | | |
