# Supplementary material for: The Public’s Awareness of and Attitude Toward Research Biobanks – A Regional German Survey
Source: Front Genet. 2018 May 24;9:190. doi: 10.3389/fgene.2018.00190 (PMC5977155; doi:10.3389/fgene.2018.00190)
Supplement: TABLE S2 — Response rate by nationality. [file Table_2.docx]

Supplementary table S2: response rate (survey) by nationality

|  | **n (persons available)** | **response rate** | |
| --- | --- | --- | --- |
|  |  | **% by nationality** | **(n)** |
| **German** | 762 | 23.8 | (181) |
| **German and other** | 86 | 10.5 | (9) |
| **other** | 150 | 9.3 | (14) |
| **total** | 998 | 20.4 | (204) |
| Differences in response rates tested by means of chi2-test; χ2=21,784, p=0.000 (two-sided); N=998 | | | |
