# Supplementary material for: The Public’s Awareness of and Attitude Toward Research Biobanks – A Regional German Survey
Source: Front Genet. 2018 May 24;9:190. doi: 10.3389/fgene.2018.00190 (PMC5977155; doi:10.3389/fgene.2018.00190)
Supplement: TEXT S1 — List of questions included in the questionnaire. [file Presentation_1.pdf]

List of survey questions (English translation by the Authors (SB))

No.	Original Question (German)	Original Categories (German)	English Question (translation)	English Categories (translation)	Source
**Attitude towards and experience with (medical) research**					
1	Was halten Sie ganz allgemein von der medizinischen Forschung?	– Ich befürworte sie eindeutig<br>– Ich befürworte sie eher<br>– Ich lehne sie eher ab<br>– Ich lehne sie eindeutig ab<br>– Unsicher	In general, what do you think of medical research?	– I definitely approve<br>– I somewhat approve<br>– I somewhat disapprove<br>– I definitely disapprove<br>– Not sure	Own question (pretested in qualitative interviews)
2	Jede Art der Forschung hat Chancen und Risiken. Wenn Sie an die medizinische Forschung denken, was meinen Sie?	– Die Chancen überwiegen klar<br>– Die Chancen überwiegen eher<br>– Die Risiken überwiegen eher<br>– Die Risiken überwiegen klar<br>– Chancen und Risiken sind gleich groß	Every kind of research has its potential benefits and risks. Thinking of medical research, what do you think?	– Benefits clearly outweigh risks<br>– Benefits somewhat outweigh risks<br>– Risks somewhat outweigh benefits<br>– Risks clearly outweigh benefits<br>– Benefits equate to risks	Own question (pretested in qualitative interviews)
3	Was halten Sie ganz allgemein von der Genforschung (als einem Gebiet der medizinischen Forschung)?	– Ich befürworte sie eindeutig<br>– Ich befürworte sie eher<br>– Ich lehne sie eher ab<br>– Ich lehne sie eindeutig ab<br>– Unsicher	In general, what do you think of genetic research (one area of medical research)?	– I definitely approve<br>– I somewhat approve<br>– I somewhat disapprove<br>– I definitely disapprove<br>– Not sure	Own question (pretested in qualitative interviews)
4	Wenn Sie an die Genforschung denken, was meinen Sie?	– Die Chancen überwiegen klar<br>– Die Chancen überwiegen eher<br>– Die Risiken überwiegen eher<br>– Die Risiken überwiegen klar<br>– Chancen und Risiken sind gleich groß	Every kind of research has its potential benefits and risks. Thinking of medical research, what do you think?	– Benefits clearly outweigh risks<br>– Benefits somewhat outweigh risks<br>– Risks somewhat outweigh benefits<br>– Risks clearly outweigh benefits<br>– Benefits equate to risks	Own question (pretested in qualitative interviews)
5	Haben Sie schon einmal an einer Studie im Bereich der medizinischen Forschung oder der Genforschung teilgenommen?	– Ja, (inkl. Angabe ungefähre Zeit und Thema)<br>– Nein, ich bin zwar gefragt worden, wollte aber nicht teilnehmen<br>– Nein, ich bin noch nie danach gefragt worden	Have you ever participated in a study in the area of medical or genetic research?	– Yes (incl. statement regarding approx. time and topic)<br>– No, I have been invited but did not want to participate.<br>– No, I have never been invited	Own question (pretested in qualitative interviews)
6	Hat eine Ihnen nahestehende Person schon einmal an einer Studie im Bereich der medizinischen Forschung oder der Genforschung teilgenommen?	– Ja (inkl. Angabe ungefähre Zeit und Thema)<br>– Nein, noch nie	Has a person close to you ever participated in a study in the area of medical or genetic research?	– Yes (incl. statement regarding approx. time and topic)<br>– No, never	Own question (pretested in qualitative interviews)
7	Haben Sie selbst schon einmal an	– Ja (inkl. Angabe ungefähre Zeit und	Have you ever participated in a	– Yes (incl. statement regarding	Own question

	einer Studie in einem anderen wissenschaftlichen Bereich teilgenommen?	Thema)<br>– Nein, noch nie	study in another area of research?	approx. time and topic)<br>– No, never	(pretested in qualitative interviews)
**Questions regarding research biobanks**					
	Einführung für nächsten Fragenblock (Fragen 8-10):<br>Als nächstes werden wir Ihnen einige Fragen zum Thema „Biobanken“ stellen. In einer Biobank werden menschliche Biomaterialien (z. B. Blut-, Urin- oder Gewebeproben) mit ausgewählten persönlichen und medizinischen Daten (z. B. Alter, Geschlecht, Blutwerte, Krankengeschichte) verknüpft und langfristig aufbewahrt. Patienten können Biomaterialien zur Verfügung zu stellen, wenn bei der Behandlung im Krankenhaus Proben übrig geblieben sind. Biobanken laden aber auch gezielt Personen zur Spende von Biomaterialien ein. Die Biomaterialien und medizinischen Daten werden in der Forschung verwendet, um die Vorbeugung, Erkennung und Behandlung von Erkrankungen zu verbessern.		Introduction of following section (questions 8-10):<br>In the following section, we are going to ask you some questions regarding “biobanks”. Biobanks collect human biomaterials (e.g. blood, urine or tissue samples), link them with selected personal and medical data (e.g. age, gender, blood values, clinical history) and store them for an indefinite period of time.<br>Patients can either provide samples when biomaterials are left over after medical treatment in a hospital, or they can directly donate biomaterials to a biobank. The biomaterials and data in biobanks are used in medical research projects aiming to improve prevention, diagnosis and treatment.		Own text adapted from the 2010 Eurobarometer questionnaire
8	Wie bewerten Sie spontan Biobanken als ein Mittel der medizinischen Forschung?	– Eindeutig positiv<br>– Eher positiv<br>– Eher negativ<br>– Eindeutig negativ<br>– Unsicher	How would you spontaneously assess biobanks	– Definitely positive<br>– Somewhat positive<br>– Somewhat negative<br>– Definitely negative<br>– Not sure	Own question (pretested in qualitative interviews)
8	Haben Sie vor dem heutigen Tag schon einmal etwas über Biobanken gehört?	– Ja<br>– Nein	Before today, have you ever heard anything about biobanks?	– Yes<br>– No	Eurobarometer 2010 (validated item)
9	Falls „Ja“, haben Sie schon einmal…<br>… vor dem heutigen Tag mit jemandem über Biobanken gesprochen?<br>… nach Informationen zu Biobanken gesucht?<br>… Material und/oder persönliche Daten für eine Biobank zur Verfügung gestellt?	– Ja, häufiger<br>– Ja, gelegentlich<br>– Ja, nur ein- oder zweimal<br>– Nein, niemals	If you have heard of biobanks: have you ever…<br>… talked about biobanks with anyone?<br>… searched for information about biobanks?<br>… donated biomaterial yourself?	– Yes, frequently<br>– Yes, occasionally<br>– Yes, only once or twice<br>– No, never	(Except for Item regarding biobank donation:) Eurobarometer 2010 (validated items)
10	Wären Sie bereit, Gewebe- oder Blutproben für eine Biobank zur Verfügung zu stellen, wenn diese bei einer Behandlung im Krankenhaus übrig geblieben	– Ja, sicher<br>– Ja, wahrscheinlich<br>– Nein, wahrscheinlich nicht<br>– Nein, sicher nicht<br>– Unsicher	Would you be willing to donate tissue or blood samples to a biobank, if those materials were left over after medical treatment in hospital? The samples would	– Yes, certainly<br>– Yes, probably<br>– No, probably not<br>– No, certainly not<br>– Not sure	Own question adapted from 2010 Eurobarometer

	sind? Die Proben wären mit einigen persönlichen Daten (z. B. Alter, Geschlecht) und mit ausgewählten medizinischen Daten (z. B. Blutwerte) verknüpft.		be linked with some personal data (e.g. your age, gender) and with selected medical data (e.g. blood values).		
**Socio-demographics**					
11	In welchem Jahr wurden Sie geboren?	– Freitext		– Free text	Own question in accordance with German standards for surveying socio-demographics
12	Sie sind…	– … male<br>– … female<br>– … other	You are…	– … male<br>– … female<br>– … other	
13	Welchen höchsten Schulabschluss haben Sie?	– Gehe noch zur Schule<br>– Von der Schule abgegangen ohne Hauptschulabschluss<br>– Haupt-/Volksschulabschluss<br>– Realschulabschluss (Mittlere Reife)<br>– Polytechnische Oberschule der DDR<br>– Fachhochschulreife/Abschluss einer Fachoberschule<br>– Abitur/allgemeine oder fachgebundene Hochschulreife<br>– Einen anderen Schulabschluss: „Freitext“	School education (highest degree)	– Still going to school<br>– Left school without diploma<br>– Secondary school qualification<br>– High-school diploma<br>– Ten-class general educational polytechnic secondary school (former Federal German Republic)<br>– University of applied sciences entrance qualification<br>– General or subject-linked university entrance qualification<br>– Another degree: “free text”	
14	Welche Ausbildungsabschlüsse haben Sie? Was alles auf dieser Liste trifft auf Sie zu?	– Befinde mich noch in Ausbildung / Studium<br>– Keinen beruflichen Abschluss und derzeit nicht in beruflicher Ausbildung<br>– Beruflich-betriebliche Berufsausbildung abgeschlossen (Lehre)<br>– Beruflich-schulische Ausbildung abgeschlossen<br>– Abschluss einer Fach-, Meister-, Technikerschule, Berufs- oder Fachakademie<br>– Bachelor an einer (Fach-)Hochschule<br>– Fachhochschulabschluss (z.B. Diplom, Master)	Professional/academic education (degrees)	– Still studying / on professional training<br>– No professional education<br>– Industrial training<br>– Graduated from Vocational academy<br>– Graduated from technical high school<br>– Bachelor from a university of applied sciences<br>– Diploma/Master from a university of applied sciences<br>– University degree (Diploma, Magister, Master, state	

		– Universitätsabschluss (z.B. Diplom, Magister, Staatsexamen)<br>– Promotion<br>– Einen anderen Ausbildungsabschluss: „Freitext“		examination)<br>– Doctorate<br>– Another education: “free text”	
15	Welche Erwerbssituation passt für Sie? Was alles auf der Liste trifft auf Sie zu?	– Vollzeiterwerbstätig<br>– Teilzeiterwerbstätig<br>– Altersteilzeit (Arbeits- oder Freistellungsphase)<br>– Geringfügig erwerbstätig, 450-Euro-Job<br>– „Ein-Euro-Job“<br>– Gelegentlich oder unregelmäßig beschäftigt<br>– In beruflicher Ausbildung/Lehre<br>– Mutterschafts-, Erziehungsurlaub, Elternzeit<br>– In Umschulung oder Weiterbildung<br>– Schule/Studium<br>– Vorruhestand, Erwerbsminderungsrente<br>– Altersrente/Pension<br>– Arbeitslosigkeit<br>– Anderes: „Freitext“	What employment status best describes your actual situation?	– Full-time<br>– Part-time<br>– Partial retirement<br>– Minor employment (up to 450 EUR per month)<br>– “One-euro-job” (work scheme for persons after long periods of unemployment)<br>– Occasional or irregular employment<br>– On vocational training<br>– On parental leave<br>– On retraining/development<br>– Still studying<br>– Early retirement<br>– Retired<br>– Unemployed<br>– Other: “free text”	
16	Haben Sie jemals eine Tätigkeit im Gesundheitswesen ausgeübt?	– Ja, früher<br>– Ja, derzeit<br>– Nein, noch nie	Have you ever professionally worked in the health care sector?	– Yes, earlier<br>– Yes, at the moment<br>– No, never	
17	Ihre Nationalität ist…?	– Deutsch<br>– Eine andere: „Freitext“	What is your nationality?	– German<br>– Another: “free text”	
