# Supplementary material for: The Public’s Awareness of and Attitude Toward Research Biobanks – A Regional German Survey
Source: Front Genet. 2018 May 24;9:190. doi: 10.3389/fgene.2018.00190 (PMC5977155; doi:10.3389/fgene.2018.00190)
Supplement: SPSS OUTPUT S1 — Binary logistic regression analyses. [file Presentation_2.pdf]

Logistic Regression; dependent variable: "prior awareness of biobanks"
Method: ENTER

Notes

Output Created		l-2017 13:14:04
Comments		
	N of Rows in Working Data File	204
Missing Value Handling	Definition of Missing	User-defined missing values are treated as missing
Syntax		LOGISTIC REGRESSION VARIABLES var_9adicho<br>/METHOD=ENTER var_3dicho age_gr Var_12 edu_grob health_job research_exp nat_dicho<br>/CONTRAST (var_3dicho)=Indicator<br>/CONTRAST (age_gr)=Indicator(1)<br>/CONTRAST (Var_12)=Indicator(1)<br>/CONTRAST (edu_grob)=Indicator(1)<br>/CONTRAST (health_job)=Indicator(1)<br>/CONTRAST (research_exp)=Indicator(1)<br>/CONTRAST (nat_dicho)=Indicator(1)<br>/PRINT=GOODFIT<br>/CRITERIA=PIN(0.05) POUT(0.10) ITERATE(20) CUT(0.5).

Case Processing Summary

Unweighted Cases[a]		N	Percent
Selected Cases	Included in Analysis	193	94,6
	Missing Cases	11	5,4
	Total	204	100,0
Unselected Cases		0	0,0
Total		204	100,0

a. If weight is in effect, see classification table for the total number of cases.

Dependent Variable Encoding

Original Value	Internal Value
0 no	0
1 yes	1

Categorical Variables Codings

		Frequency	Parameter coding (1)	(2)	(3)	(4)	(5)	(6)
age_gr age-groups (steps of 10 years)	1 18-29	31	0,000	0,000	0,000	0,000	0,000	0,000
	2 30-39	18	1,000	0,000	0,000	0,000	0,000	0,000
	3 40-49	33	0,000	1,000	0,000	0,000	0,000	0,000
	4 50-59	46	0,000	0,000	1,000	0,000	0,000	0,000
	5 60-69	22	0,000	0,000	0,000	1,000	0,000	0,000
	6 70-79	31	0,000	0,000	0,000	0,000	1,000	0,000
	7 80 oder älter	12	0,000	0,000	0,000	0,000	0,000	1,000
edu_grob school education (low - middle - high)	1 low (kein Abschluss/Hauptschule)	24	0,000	0,000	0,000			
	2 middle (Realschule)	46	1,000	0,000	0,000			
	3 high ((Fach)-Abitur)	113	0,000	1,000	0,000			
	8 other/still going to school	10	0,000	0,000	1,000			
var_3dicho assessment of genetic research (approve - disapprove - not sure)	0 disapprove (somewhat/definitely)	33	1,000	0,000				
	1 approve (somewhat/definitely)	121	0,000	1,000				
	3 not sure	39	0,000	0,000				
health_job have you ever worked in the health care-sector?	0 no, never	155	0,000					
	1 yes, currently/before	38	1,000					
research_exp have you ever participated in research?	0 no	165	0,000					
	1 yes	28	1,000					
Var_12 gender	1 male	82	0,000					
	2 female	111	1,000					
nat_dicho nationality (binary coding)	1 only German	178	0,000					
	2 German & other / only other	15	1,000					

Block 0: Beginning Block

Classification Table[a,b]

Observed			Predicted var_9adicho ever heard of biobanks before (0/1-coding) 0 no	1 yes	Percentage Correct
Step 0	var_9adicho ever heard of biobanks before (0/1-coding)	0 no	133	0	100,0
		1 yes	60	0	0,0
	Overall Percentage				68,9

a. Constant is included in the model.

b. The cut value is ,500

Variables in the Equation

		B	S.E.	Wald	df	Sig.	Exp(B)
Step 0	Constant	-0,796	0,156	26,199	1	0,000	0,451

Variables not in the Equation

			Score	df	Sig.
Step 0	Variables	assessment of genetic research (approve - disapprove - not sure)	0,300	2	0,861
		assessment of genetic research (approve - disapprove - not sure)(1)	0,270	1	0,603
		assessment of genetic research (approve - disapprove - not sure)(2)	0,198	1	0,656
		age-groups (steps of 10 years)	3,571	6	0,735
		age-groups (steps of 10 years)(1)	0,047	1	0,829
		age-groups (steps of 10 years)(2)	0,270	1	0,603
		age-groups (steps of 10 years)(3)	1,823	1	0,177
		age-groups (steps of 10 years)(4)	0,323	1	0,570
		age-groups (steps of 10 years)(5)	0,073	1	0,787
		age-groups (steps of 10 years)(6)	1,242	1	0,265
		gender(1)	0,225	1	0,635
		school education (low - middle - high)	12,276	3	0,006
		school education (low - middle - high)(1)	0,705	1	0,401
		school education (low - middle - high)(2)	4,704	1	0,030
		school education (low - middle - high)(3)	1,761	1	0,185
		have you ever worked in the health care-sector?(1)	0,215	1	0,643
		have you ever participated in research?(1)	2,118	1	0,146
		nationality (binary coding)(1)	0,603	1	0,437
	Overall Statistics		19,756	15	0,182

Block 1: Method = Enter

Omnibus Tests of Model Coefficients

		Chi-square	df	Sig.
Step 1	Step	23,582	15	0,073
	Block	23,582	15	0,073
	Model	23,582	15	0,073

Model Summary

Step	-2 Log likelihood	Cox & Snell R Square	Nagelkerke R Square
1	215,662[a]	0,115	0,162

a. Estimation terminated at iteration number 6 because parameter estimates changed by less [illegible]

Hosmer and Lemeshow Test

Step	Chi-square	df	Sig.
1	5,415	8	0,712

Contingency Table for Hosmer and Lemeshow Test

		var_9adicho ever heard of biobanks before (0/1-coding) = 0 no		var_9adicho ever heard of biobanks before (0/1-coding) = 1 yes		
		Observed	Expected	Observed	Expected	Total
Step 1	1	19	18,339	0	0,661	19
	2	16	16,738	3	2,262	19
	3	12	14,685	7	4,315	19
	4	15	15,344	6	5,656	21
	5	15	13,364	4	5,636	19
	6	13	12,700	6	6,300	19
	7	14	11,963	5	7,037	19
	8	11	11,572	9	8,428	20
	9	11	10,005	8	8,995	19
	10	7	8,290	12	10,710	19

Classification Table[a]

Observed			Predicted: var_9adicho ever heard of biobanks before (0/1-coding)		Percentage Correct
			0 no	1 yes	
Step 1	var_9adicho ever heard of biobanks before (0/1-coding)	0 no	124	9	93,2
		1 yes	48	12	20,0
	Overall Percentage				70,5

a. The cut value is ,500

Variables in the Equation

		B	S.E.	Wald	df	Sig.	Exp(B)
Step 1[a]	assessment of genetic research (approve - disapprove - not sure)			1,624	2	0,444	
	assessment of genetic research (approve - disapprove - not sure)(1)	-0,748	0,599	1,558	1	0,212	0,474
	assessment of genetic research (approve - disapprove - not sure)(2)	-0,420	0,449	0,877	1	0,349	0,657
	age-groups (steps of 10 years)			5,576	6	0,472	
	age-groups (steps of 10 years)(1)	0,340	0,666	0,261	1	0,609	1,406
	age-groups (steps of 10 years)(2)	0,277	0,590	0,221	1	0,639	1,320
	age-groups (steps of 10 years)(3)	0,999	0,563	3,147	1	0,076	2,716
	age-groups (steps of 10 years)(4)	0,992	0,659	2,269	1	0,132	2,697
	age-groups (steps of 10 years)(5)	0,726	0,633	1,315	1	0,252	2,067
	age-groups (steps of 10 years)(6)	-0,314	0,924	0,116	1	0,734	0,730
	gender(1)	-0,239	0,348	0,472	1	0,492	0,788
	school education (low - middle - high)			9,421	3	0,024	
	school education (low - middle - high)(1)	2,229	1,094	4,149	1	0,042	9,286
	school education (low - middle - high)(2)	2,883	1,072	7,231	1	0,007	17,866
	school education (low - middle - high)(3)	3,270	1,236	7,003	1	0,008	26,320
	have you ever worked in the health care-sector?(1)	0,127	0,427	0,088	1	0,767	1,135
	have you ever participated in research?(1)	0,443	0,461	0,922	1	0,337	1,557
	nationality (binary coding)(1)	0,427	0,610	0,492	1	0,483	1,533
	Constant	-3,503	1,155	9,192	1	0,002	0,030

a. Variable(s) entered on step 1: assessment of genetic research (approve - disapprove - not sure), age-groups (steps of 10 years), gender, school education (low - middle - high), have you ever [illegible]

Logistic Regression; dependent variable: "prior awareness of biobanks"
Method: Forward Stepwise (Wald)

Notes

Output Created		l-2017 13:14:04
	N of Rows in Working Data File	204
Missing Value Handling	Definition of Missing	User-defined missing values are treated as missing
Syntax		LOGISTIC REGRESSION VARIABLES var_9adicho<br>/METHOD=FSTEP(WALD) var_3dicho age_gr Var_12 edu_grob health_job research_exp nat_dicho<br>/CONTRAST (var_3dicho)=Indicator<br>/CONTRAST (age_gr)=Indicator(1)<br>/CONTRAST (Var_12)=Indicator(1)<br>/CONTRAST (edu_grob)=Indicator(1)<br>/CONTRAST (health_job)=Indicator(1)<br>/CONTRAST (research_exp)=Indicator(1)<br>/CONTRAST (nat_dicho)=Indicator(1)<br>/PRINT=GOODFIT<br>/CRITERIA=PIN(0.05) POUT(0.10) ITERATE(20) CUT(0.5).

Case Processing Summary

Unweighted Cases[a]		N	Percent
Selected Cases	Included in Analysis	193	94,6
	Missing Cases	11	5,4
	Total	204	100,0
Unselected Cases		0	0,0
Total		204	100,0

a. If weight is in effect, see classification table for the total number of cases.

Dependent Variable Encoding

Original Value	Internal Value
0 no	0
1 yes	1

Categorical Variables Codings

		Frequency	Parameter coding (1)	(2)	(3)	(4)	(5)	(6)
age_gr age-groups (steps of 10 years)	1 18-29	31	0,000	0,000	0,000	0,000	0,000	0,000
	2 30-39	18	1,000	0,000	0,000	0,000	0,000	0,000
	3 40-49	33	0,000	1,000	0,000	0,000	0,000	0,000
	4 50-59	46	0,000	0,000	1,000	0,000	0,000	0,000
	5 60-69	22	0,000	0,000	0,000	1,000	0,000	0,000
	6 70-79	31	0,000	0,000	0,000	0,000	1,000	0,000
	7 80 oder älter	12	0,000	0,000	0,000	0,000	0,000	1,000
edu_grob school education (low - middle - high)	1 low (kein Abschluss/Hauptschule)	24	0,000	0,000	0,000			
	2 middle (Realschule)	46	1,000	0,000	0,000			
	3 high ((Fach)-Abitur)	113	0,000	1,000	0,000			
	8 other/still going to school	10	0,000	0,000	1,000			
var_3dicho assessment of genetic research (approve - disapprove - not sure)	0 disapprove (somewhat/definitely)	33	1,000	0,000				
	1 approve (somewhat/definitely)	121	0,000	1,000				
	3 not sure	39	0,000	0,000				
health_job have you ever worked in the health care-sector?	0 no, never	155	0,000					
	1 yes, currently/before	38	1,000					
research_exp have you ever participated in research?	0 no	165	0,000					
	1 yes	28	1,000					
Var_12 gender	1 male	82	0,000					
	2 female	111	1,000					
nat_dicho nationality (binary coding)	1 only German	178	0,000					
	2 German & other / only other	15	1,000					

Block 0: Beginning Block

Classification Table[a,b]

Observed			Predicted: var_9adicho ever heard of biobanks before (0/1-coding) 0 no	1 yes	Percentage Correct
Step 0	var_9adicho ever heard of biobanks before (0/1-coding)	0 no	133	0	100,0
		1 yes	60	0	0,0
	Overall Percentage				68,9

a. Constant is included in the model.

b. The cut value is ,500

Variables in the Equation

		B	S.E.	Wald	df	Sig.	Exp(B)
Step 0	Constant	-0,796	0,156	26,199	1	0,000	0,451

Variables not in the Equation

			Score	df	Sig.
Step 0	Variables	assessment of genetic research (approve - disapprove - not sure)	0,300	2	0,861
		assessment of genetic research (approve - disapprove - not sure)(1)	0,270	1	0,603
		assessment of genetic research (approve - disapprove - not sure)(2)	0,198	1	0,656
		age-groups (steps of 10 years)	3,571	6	0,735
		age-groups (steps of 10 years)(1)	0,047	1	0,829
		age-groups (steps of 10 years)(2)	0,270	1	0,603
		age-groups (steps of 10 years)(3)	1,823	1	0,177
		age-groups (steps of 10 years)(4)	0,323	1	0,570
		age-groups (steps of 10 years)(5)	0,073	1	0,787
		age-groups (steps of 10 years)(6)	1,242	1	0,265
		gender(1)	0,225	1	0,635
		school education (low - middle - high)	12,276	3	0,006
		school education (low - middle - high)(1)	0,705	1	0,401
		school education (low - middle - high)(2)	4,704	1	0,030
		school education (low - middle - high)(3)	1,761	1	0,185
		have you ever worked in the health care-sector?(1)	0,215	1	0,643
		have you ever participated in research?(1)	2,118	1	0,146
		nationality (binary coding)(1)	0,603	1	0,437
	Overall Statistics		19,756	15	0,182

Block 1: Method = Forward Stepwise (Wald)

Omnibus Tests of Model Coefficients

		Chi-square	df	Sig.
Step 1	Step	15,138	3	0,002
	Block	15,138	3	0,002
	Model	15,138	3	0,002

Model Summary

Step	-2 Log likelihood	Cox & Snell R Square	Nagelkerke R Square
1	224,106[a]	0,075	0,106

a. Estimation terminated at iteration number 6 because parameter estimates changed by less than ,001.

Hosmer and Lemeshow Test

Step	Chi-square	df	Sig.
1	0,000	2	1,000

Contingency Table for Hosmer and Lemeshow Test

		var_9adicho ever heard of biobanks before (0/1-coding) = 0 no		var_9adicho ever heard of biobanks before (0/1-coding) = 1 yes		Total
		Observed	Expected	Observed	Expected	
Step 1	1	23	23,000	1	1,000	24
	2	34	34,000	12	12,000	46
	3	71	71,000	42	42,000	113
	4	5	5,000	5	5,000	10

Classification Table[a]

Observed			Predicted: var_9adicho ever heard of biobanks before (0/1-coding) 0 no	Predicted: var_9adicho ever heard of biobanks before (0/1-coding) 1 yes	Percentage Correct
Step 1	var_9adicho ever heard of biobanks before (0/1-coding)	0 no	128	5	96,2
		1 yes	55	5	8,3
	Overall Percentage				68,9

a. The cut value is ,500

Variables in the Equation

		B	S.E.	Wald	df	Sig.	Exp(B)
Step 1[a]	school education (low - middle - high)			8,726	3	0,033	
	school education (low - middle - high)(1)	2,094	1,075	3,793	1	0,051	8,118
	school education (low - middle - high)(2)	2,610	1,040	6,302	1	0,012	13,606
	school education (low - middle - high)(3)	3,135	1,201	6,811	1	0,009	23,000
	Constant	-3,135	1,022	9,422	1	0,002	0,043

a. Variable(s) entered on step 1: school education (low - middle - high).

Variables not in the Equation

			Score	df	Sig.
Step 1	Variables	assessment of genetic research (approve - disapprove - not sure)	0,761	2	0,683
		assessment of genetic research (approve - disapprove - not sure)(1)	0,667	1	0,414
		assessment of genetic research (approve - disapprove - not sure)(2)	0,068	1	0,794
		age-groups (steps of 10 years)	5,066	6	0,535
		age-groups (steps of 10 years)(1)	0,036	1	0,850
		age-groups (steps of 10 years)(2)	0,402	1	0,526
		age-groups (steps of 10 years)(3)	2,476	1	0,116
		age-groups (steps of 10 years)(4)	0,567	1	0,451
		age-groups (steps of 10 years)(5)	0,085	1	0,770
		age-groups (steps of 10 years)(6)	1,003	1	0,317
		gender(1)	0,419	1	0,517
		have you ever worked in the health care-sector?(1)	0,121	1	0,728
		have you ever participated in research?(1)	0,806	1	0,369
		nationality (binary coding)(1)	0,184	1	0,668
	Overall Statistics		8,146	12	0,774

Logistic Regression; dependent variable: "assessment of biobanks"
Method: ENTER

Notes

Output Created		l-2017 13:14:04
Comments		
N of Rows in Working Data File		204
Missing Value Handling	Definition of Missing	User-defined missing values are treated as missing
Syntax		LOGISTIC REGRESSION VARIABLES var_8dicho<br>/METHOD=ENTER var_3dicho age_gr Var_12 edu_grob health_job research_exp nat_dicho var_9adicho<br>/CONTRAST (var_3dicho)=Indicator<br>/CONTRAST (age_gr)=Indicator(1)<br>/CONTRAST (Var_12)=Indicator(1)<br>/CONTRAST (edu_grob)=Indicator(1)<br>/CONTRAST (health_job)=Indicator(1)<br>/CONTRAST (research_exp)=Indicator(1)<br>/CONTRAST (nat_dicho)=Indicator(1)<br>/CONTRAST (var_9adicho)=Indicator(1)<br>/PRINT=GOODFIT<br>/CRITERIA=PIN(0.05) POUT(0.10) ITERATE(20) CUT(0.5).
Resources	Processor Time	00:00:00,05
	Elapsed Time	00:00:00,04

Case Processing Summary

Unweighted Cases[a]		N	Percent
Selected Cases	Included in Analysis	165	80,9
	Missing Cases	39	19,1
	Total	204	100,0
Unselected Cases		0	0,0
Total		204	100,0

a. If weight is in effect, see classification table for the total number of cases.

Dependent Variable Encoding

Original Value	Internal Value
1 definitely/somewhat positive	0
2 definitely/somewhat negative	1

Categorical Variables Codings

		Frequency	Parameter coding (1)	(2)	(3)	(4)	(5)	(6)
age_gr age-groups (steps of 10 years)	1 18-29	26	0,000	0,000	0,000	0,000	0,000	0,000
	2 30-39	15	1,000	0,000	0,000	0,000	0,000	0,000
	3 40-49	25	0,000	1,000	0,000	0,000	0,000	0,000
	4 50-59	43	0,000	0,000	1,000	0,000	0,000	0,000
	5 60-69	17	0,000	0,000	0,000	1,000	0,000	0,000
	6 70-79	27	0,000	0,000	0,000	0,000	1,000	0,000
	7 80 oder älter	12	0,000	0,000	0,000	0,000	0,000	1,000
edu_grob school education (low - middle - high)	1 low (kein Abschluss/Hauptschule)	19	0,000	0,000	0,000			
	2 middle (Realschule)	40	1,000	0,000	0,000			
	3 high ((Fach)-Abitur)	96	0,000	1,000	0,000			
	8 other/still going to school	10	0,000	0,000	1,000			
var_3dicho assessment of genetic research (approve - disapprove - not sure)	0 disapprove (somewhat/definitely)	21	1,000	0,000				
	1 approve (somewhat/definitely)	110	0,000	1,000				
	3 not sure	34	0,000	0,000				
health_job have you ever worked in the health care-sector?	0 no, never	132	0,000					
	1 yes, currently/before	33	1,000					
nat_dicho nationality (binary coding)	1 only German	151	0,000					
	2 German & other / only other	14	1,000					
Var_12 gender	1 male	76	0,000					
	2 female	89	1,000					
research_exp have you ever participated in research?	0 no	139	0,000					
	1 yes	26	1,000					
var_9adicho ever heard of biobanks before (0/1-coding)	0 no	108	0,000					
	1 yes	57	1,000					

Block 0: Beginning Block

Classification Table[a,b]

Observed			Predicted: var_8dicho spontaneous assessment of biobanks (positive - negative - not sure): 1 definitely/somewhat positive	2 definitely/somewhat negative	Percentage Correct
Step 0	var_8dicho spontaneous assessment of biobanks (positive - negative - not sure)	1 definitely/somewhat positive	155	0	100,0
		2 definitely/somewhat negative	10	0	0,0
	Overall Percentage				93,9

a. Constant is included in the model.

b. The cut value is ,500

Variables in the Equation

		B	S.E.	Wald	df	Sig.	Exp(B)
Step 0	Constant	-2,741	0,326	70,569	1	0,000	0,065

Variables not in the Equation

			Score	df	Sig.
Step 0	Variables	assessment of genetic research (approve - disapprove - not sure)	13,336	2	0,001
		assessment of genetic research (approve - disapprove - not sure)(1)	13,314	1	0,000
		assessment of genetic research (approve - disapprove - not sure)(2)	3,406	1	0,065
		age-groups (steps of 10 years)	7,775	6	0,255
		age-groups (steps of 10 years)(1)	0,011	1	0,918
		age-groups (steps of 10 years)(2)	0,195	1	0,659
		age-groups (steps of 10 years)(3)	0,086	1	0,770
		age-groups (steps of 10 years)(4)	4,469	1	0,035
		age-groups (steps of 10 years)(5)	2,083	1	0,149
		age-groups (steps of 10 years)(6)	0,117	1	0,732
		gender(1)	0,066	1	0,797
		school education (low - middle - high)	3,260	3	0,353
		school education (low - middle - high)(1)	1,176	1	0,278
		school education (low - middle - high)(2)	2,083	1	0,149
		school education (low - middle - high)(3)	0,290	1	0,590
		have you ever worked in the health care-sector?(1)	0,665	1	0,415
		have you ever participated in research?(1)	0,144	1	0,704
		nationality (binary coding)(1)	1,818	1	0,178
		ever heard of biobanks before (0/1-coding)(1)	1,124	1	0,289
	Overall Statistics		23,344	16	0,105

Block 1: Method = Enter

Omnibus Tests of Model Coefficients

		Chi-square	df	Sig.
Step 1	Step	24,098	16	0,087
	Block	24,098	16	0,087
	Model	24,098	16	0,087

Model Summary

Step	-2 Log likelihood	Cox & Snell R	Nagelkerke R
1	51,350[a]	0,136	0,370

a. Estimation terminated at iteration number 20 because maximum iterations has been reached.

Hosmer and Lemeshow Test

Step	Chi-square	df	Sig.
1	3,320	8	0,913

Contingency Table for Hosmer and Lemeshow Test

		var_8dicho spontaneous		var_8dicho spontaneous		Total
		Observed	Expected	Observed	Expected	
Step 1	1	17	17,000	0	0,000	17
	2	17	17,000	0	0,000	17
	3	17	17,000	0	0,000	17
	4	16	15,987	0	0,013	16
	5	17	16,826	0	0,174	17
	6	16	16,589	1	0,411	17
	7	16	16,343	1	0,657	17
	8	18	16,699	0	1,301	18
	9	13	14,147	4	2,853	17
	10	8	7,409	4	4,591	12

Classification Table[a]

Observed			Predicted: var_8dicho spontaneous assessment of biobanks (positive - negative - not sure) 1 definitely/somewhat positive	2 definitely/somewhat negative	Percentage Correct
Step 1	var_8dicho spontaneous assessment of biobanks (positive - negative - not sure)	1 definitely/somewhat positive	154	1	99,4
		2 definitely/somewhat negative	9	1	10,0
	Overall Percentage				93,9

a. The cut value is ,500

Variables in the Equation

		B	S.E.	Wald	df	Sig.	Exp(B)
Step 1[a]	assessment of genetic research (approve - disapprove - not sure)			6,722	2	0,035	
	assessment of genetic research (approve - disapprove - not sure)(1)	2,252	1,413	2,542	1	0,111	9,508
	assessment of genetic research (approve - disapprove - not sure)(2)	-0,292	1,312	0,050	1	0,824	0,746
	age-groups (steps of 10 years)			1,805	6	0,937	
	age-groups (steps of 10 years)(1)	18,893	7426,423	0,000	1	0,998	160418887,573
	age-groups (steps of 10 years)(2)	18,514	7426,423	0,000	1	0,998	109791003,304
	age-groups (steps of 10 years)(3)	18,470	7426,423	0,000	1	0,998	105045911,031
	age-groups (steps of 10 years)(4)	19,637	7426,423	0,000	1	0,998	337328226,188
	age-groups (steps of 10 years)(5)	0,098	9989,762	0,000	1	1,000	1,103
	age-groups (steps of 10 years)(6)	18,091	7426,423	0,000	1	0,998	71928610,676
	gender(1)	0,165	0,850	0,038	1	0,846	1,180
	school education (low - middle - high)			1,444	3	0,695	
	school education (low - middle - high)(1)	17,162	7850,261	0,000	1	0,998	28417002,799
	school education (low - middle - high)(2)	18,632	7850,261	0,000	1	0,998	123580746,167
	school education (low - middle - high)(3)	18,497	7850,261	0,000	1	0,998	107905731,828
	have you ever worked in the health care-sector?(1)	1,403	0,990	2,010	1	0,156	4,068
	have you ever participated in research?(1)	0,241	1,094	0,048	1	0,826	1,272
	nationality (binary coding)(1)	0,051	1,075	0,002	1	0,962	1,053
	ever heard of biobanks before (0/1-coding)(1)	0,730	0,781	0,874	1	0,350	2,075
	Constant	-40,633	10806,403	0,000	1	0,997	0,000

a. Variable(s) entered on step 1: assessment of genetic research (approve - disapprove - not sure), age-groups (steps of 10 years), gender, school education (low - middle - high), have you ever worked in the health care-sector?, have you ever participated in research?, nationality (binary coding), ever heard of biobanks before (0/1-coding).

Logistic Regression; dependent variable: "assessment of biobanks"
Method: Forward Stepwise (Wald)

Notes

Output Created		l-2017 13:14:04
	N of Rows in Working Data File	204
Missing Value Handling	Definition of Missing	User-defined missing values are treated as missing
Syntax		LOGISTIC REGRESSION VARIABLES var_8dicho<br>/METHOD=FSTEP(WALD) var_3dicho age_gr Var_12 edu_grob health_job research_exp nat_dicho var_9adicho<br>/CONTRAST (var_3dicho)=Indicator<br>/CONTRAST (age_gr)=Indicator(1)<br>/CONTRAST (Var_12)=Indicator(1)<br>/CONTRAST (edu_grob)=Indicator(1)<br>/CONTRAST (health_job)=Indicator(1)<br>/CONTRAST (research_exp)=Indicator(1)<br>/CONTRAST (nat_dicho)=Indicator(1)<br>/CONTRAST (var_9adicho)=Indicator(1)<br>/PRINT=GOODFIT<br>/CRITERIA=PIN(0.05) POUT(0.10) ITERATE(20) CUT(0.5).
Resources	Processor Time	00:00:00,03
	Elapsed Time	00:00:00,03

Case Processing Summary

Unweighted Cases[a]		N	Percent
Selected Cases	Included in Analysis	165	80,9
	Missing Cases	39	19,1
	Total	204	100,0
Unselected Cases		0	0,0
Total		204	100,0

a. If weight is in effect, see classification table for the total number of cases.

Dependent Variable Encoding

Original Value	Internal Value
1 definitely/somewhat positive	0
2 definitely/somewhat negative	1

Categorical Variables Codings

		Frequency	Parameter coding (1)	(2)	(3)	(4)	(5)	(6)
age_gr age-groups (steps of 10 years)	1 18-29	26	0,000	0,000	0,000	0,000	0,000	0,000
	2 30-39	15	1,000	0,000	0,000	0,000	0,000	0,000
	3 40-49	25	0,000	1,000	0,000	0,000	0,000	0,000
	4 50-59	43	0,000	0,000	1,000	0,000	0,000	0,000
	5 60-69	17	0,000	0,000	0,000	1,000	0,000	0,000
	6 70-79	27	0,000	0,000	0,000	0,000	1,000	0,000
	7 80 oder älter	12	0,000	0,000	0,000	0,000	0,000	1,000
edu_grob school education (low - middle - high)	1 low (kein Abschluss/Hauptschule)	19	0,000	0,000	0,000			
	2 middle (Realschule)	40	1,000	0,000	0,000			
	3 high ((Fach)-Abitur)	96	0,000	1,000	0,000			
	8 other/still going to school	10	0,000	0,000	1,000			
var_3dicho assessment of genetic research (approve - disapprove - not sure)	0 disapprove (somewhat/definitely)	21	1,000	0,000				
	1 approve (somewhat/definitely)	110	0,000	1,000				
	3 not sure	34	0,000	0,000				
health_job have you ever worked in the health care-sector?	0 no, never	132	0,000					
	1 yes, currently/before	33	1,000					
nat_dicho nationality (binary coding)	1 only German	151	0,000					
	2 German & other / only other	14	1,000					
Var_12 gender	1 male	76	0,000					
	2 female	89	1,000					
research_exp have you ever participated in research?	0 no	139	0,000					
	1 yes	26	1,000					
var_9adicho ever heard of biobanks before (0/1-coding)	0 no	108	0,000					
	1 yes	57	1,000					

Block 0: Beginning Block

Classification Table[a,b]

Observed			Predicted: var_8dicho spontaneous assessment of biobanks (positive - negative - not sure) 1 definitely/somewhat positive	2 definitely/somewhat negative	Percentage Correct
Step 0	var_8dicho spontaneous assessment of biobanks (positive - negative - not sure)	1 definitely/somewhat positive	155	0	100,0
		2 definitely/somewhat negative	10	0	0,0
	Overall Percentage				93,9

a. Constant is included in the model.
b. The cut value is ,500

Variables in the Equation

		B	S.E.	Wald	df	Sig.	Exp(B)
Step 0	Constant	-2,741	0,326	70,569	1	0,000	0,065

Variables not in the Equation

			Score	df	Sig.
Step 0	Variables	assessment of genetic research (approve - disapprove - not sure)	13,336	2	0,001
		assessment of genetic research (approve - disapprove - not sure)(1)	13,314	1	0,000
		assessment of genetic research (approve - disapprove - not sure)(2)	3,406	1	0,065
		age-groups (steps of 10 years)	7,775	6	0,255
		age-groups (steps of 10 years)(1)	0,011	1	0,918
		age-groups (steps of 10 years)(2)	0,195	1	0,659
		age-groups (steps of 10 years)(3)	0,086	1	0,770
		age-groups (steps of 10 years)(4)	4,469	1	0,035
		age-groups (steps of 10 years)(5)	2,083	1	0,149
		age-groups (steps of 10 years)(6)	0,117	1	0,732
		gender(1)	0,066	1	0,797
		school education (low - middle - high)	3,260	3	0,353
		school education (low - middle - high)(1)	1,176	1	0,278
		school education (low - middle - high)(2)	2,083	1	0,149
		school education (low - middle - high)(3)	0,290	1	0,590
		have you ever worked in the health care-sector?(1)	0,665	1	0,415
		have you ever participated in research?(1)	0,144	1	0,704
		nationality (binary coding)(1)	1,818	1	0,178
		ever heard of biobanks before (0/1-coding)(1)	1,124	1	0,289
	Overall Statistics		23,344	16	0,105

Block 1: Method = Forward Stepwise (Wald)

Omnibus Tests of Model Coefficients

		Chi-square	df	Sig.
Step 1	Step	9,007	2	0,011
	Block	9,007	2	0,011
	Model	9,007	2	0,011

Model Summary

Step	-2 Log likelihood	Cox & Snell R Square	Nagelkerke R Square
1	66,442[a]	0,053	0,145

a. Estimation terminated at iteration number 6 because parameter estimates changed by less than ,001.

Hosmer and Lemeshow Test

Step	Chi-square	df	Sig.
1	0,000	1	1,000

Contingency Table for Hosmer and Lemeshow Test

		var_8dicho spontaneous assessment of biobanks (positive - negative - not sure) = 1 definitely/somewhat positive		var_8dicho spontaneous assessment of biobanks (positive - negative - not sure) = 2 definitely/somewhat negative		
		Observed	Expected	Observed	Expected	Total
Step 1	1	33	33,000	1	1,000	34
	2	106	106,000	4	4,000	110
	3	16	16,000	5	5,000	21

Classification Table[a]

Observed			Predicted: var_8dicho spontaneous assessment of biobanks (positive - negative - not sure)		
			1 definitely/somewhat positive	2 definitely/somewhat negative	Percentage Correct
Step 1	var_8dicho spontaneous assessment of biobanks (positive - negative - not sure)	1 definitely/somewhat positive	155	0	100,0
		2 definitely/somewhat negative	10	0	0,0
	Overall Percentage				93,9

a. The cut value is ,500

Variables in the Equation

		B	S.E.	Wald	df	Sig.	Exp(B)
Step 1[a]	assessment of genetic research (approve - disapprove - not sure)			9,952	2	0,007	
	assessment of genetic research (approve - disapprove - not sure)(1)	2,333	1,137	4,211	1	0,040	10,312
	assessment of genetic research (approve - disapprove - not sure)(2)	0,219	1,136	0,037	1	0,847	1,245
	Constant	-3,497	1,015	11,866	1	0,001	0,030

a. Variable(s) entered on step 1: assessment of genetic research (approve - disapprove - not sure).

Variables not in the Equation

			Score	df	Sig.
Step 1	Variables	age-groups (steps of 10 years)	5,031	6	0,540
		age-groups (steps of 10 years)(1)	0,499	1	0,480
		age-groups (steps of 10 years)(2)	0,099	1	0,753
		age-groups (steps of 10 years)(3)	0,139	1	0,709
		age-groups (steps of 10 years)(4)	1,809	1	0,179
		age-groups (steps of 10 years)(5)	1,500	1	0,221
		age-groups (steps of 10 years)(6)	0,063	1	0,802
		gender(1)	0,042	1	0,838
		school education (low - middle - high)	3,191	3	0,363
		school education (low - middle - high)(1)	0,946	1	0,331
		school education (low - middle - high)(2)	2,754	1	0,097
		school education (low - middle - high)(3)	0,034	1	0,853
		have you ever worked in the health care-sector?(1)	1,805	1	0,179
		have you ever participated in research?(1)	0,470	1	0,493
		nationality (binary coding)(1)	0,528	1	0,468
		ever heard of biobanks before (0/1-coding)(1)	1,723	1	0,189
	Overall Statistics		11,372	14	0,657

Logistic Regression; dependent variable: "hypothetical willingness to donate biomaterial and/or data" Method: ENTER

Notes

Output Created		l-2017 13:14:04
	N of Rows in Working Data File	204
Missing Value Handling	Definition of Missing	User-defined missing values are treated as missing
Syntax		LOGISTIC REGRESSION VARIABLES var_10dicho<br>/METHOD=ENTER var_3dicho age_gr Var_12 edu_grob health_job research_exp nat_dicho var_9adicho<br>/CONTRAST (var_3dicho)=Indicator<br>/CONTRAST (age_gr)=Indicator(1)<br>/CONTRAST (Var_12)=Indicator(1)<br>/CONTRAST (edu_grob)=Indicator(1)<br>/CONTRAST (health_job)=Indicator(1)<br>/CONTRAST (research_exp)=Indicator(1)<br>/CONTRAST (nat_dicho)=Indicator(1)<br>/CONTRAST (var_9adicho)=Indicator(1)<br>/PRINT=GOODFIT<br>/CRITERIA=PIN(0.05) POUT(0.10) ITERATE(20) CUT(0.5).
Resources	Processor Time	00:00:00,03
	Elapsed Time	00:00:00,05

Case Processing Summary

Unweighted Cases[a]		N	Percent
Selected Cases	Included in Analysis	169	82,8
	Missing Cases	35	17,2
	Total	204	100,0
Unselected Cases		0	0,0
Total		204	100,0

a. If weight is in effect, see classification table for the total number of cases.

Dependent Variable Encoding

Original Value	Internal Value
0 no (probably/certainly not)	0
1 yes (probably/certainly	1

Categorical Variables Codings

		Frequency	Parameter coding (1)	(2)	(3)	(4)	(5)	(6)
age_gr age-groups (steps of 10 years)	1 18-29	27	0,000	0,000	0,000	0,000	0,000	0,000
	2 30-39	17	1,000	0,000	0,000	0,000	0,000	0,000
	3 40-49	25	0,000	1,000	0,000	0,000	0,000	0,000
	4 50-59	41	0,000	0,000	1,000	0,000	0,000	0,000
	5 60-69	20	0,000	0,000	0,000	1,000	0,000	0,000
	6 70-79	29	0,000	0,000	0,000	0,000	1,000	0,000
	7 80 oder älter	10	0,000	0,000	0,000	0,000	0,000	1,000
edu_grob school education (low - middle - high)	1 low (kein Abschluss/Hauptschule)	18	0,000	0,000	0,000			
	2 middle (Realschule)	41	1,000	0,000	0,000			
	3 high ((Fach)-Abitur)	100	0,000	1,000	0,000			
	8 other/still going to school	10	0,000	0,000	1,000			
var_3dicho assessment of genetic research (approve - disapprove - not sure)	0 disapprove (somewhat/definitely)	27	1,000	0,000				
	1 approve (somewhat/definitely)	111	0,000	1,000				
	3 not sure	31	0,000	0,000				
health_job have you ever worked in the health care-sector?	0 no, never	132	0,000					
	1 yes, currently/before	37	1,000					
nat_dicho nationality (binary coding)	1 only German	157	0,000					
	2 German & other / only other	12	1,000					
Var_12 gender	1 male	73	0,000					
	2 female	96	1,000					
research_exp have you ever participated in research?	0 no	142	0,000					
	1 yes	27	1,000					
var_9adicho ever heard of biobanks before (0/1-coding)	0 no	115	0,000					
	1 yes	54	1,000					

Block 0: Beginning Block

Classification Table[a,b]

Observed			Predicted: var_10dicho willingness to participate in biobank-research (yes - no - not sure) 0 no (probably/certainly not)	1 yes (probably/certainly	Percentage Correct
Step 0	var_10dicho willingness to participate in biobank-research (yes - no - not sure)	0 no (probably/certainly not)	0	34	0,0
		1 yes (probably/certainly	0	135	100,0
	Overall Percentage				79,9

a. Constant is included in the model.

b. The cut value is ,500

Variables in the Equation

		B	S.E.	Wald	df	Sig.	Exp(B)
Step 0	Constant	1,379	0,192	51,642	1	0,000	3,971

Variables not in the Equation

			Score	df	Sig.
Step 0	Variables	assessment of genetic research (approve - disapprove - not sure)	15,754	2	0,000
		assessment of genetic research (approve - disapprove - not sure)(1)	15,710	1	0,000
		assessment of genetic research (approve - disapprove - not sure)(2)	6,548	1	0,011
		age-groups (steps of 10 years)	11,520	6	0,074
		age-groups (steps of 10 years)(1)	1,016	1	0,314
		age-groups (steps of 10 years)(2)	0,310	1	0,578
		age-groups (steps of 10 years)(3)	1,013	1	0,314
		age-groups (steps of 10 years)(4)	5,580	1	0,018
		age-groups (steps of 10 years)(5)	2,081	1	0,149
		age-groups (steps of 10 years)(6)	2,614	1	0,106
		gender(1)	3,296	1	0,069
		school education (low - middle - high)	1,418	3	0,701
		school education (low - middle - high)(1)	1,013	1	0,314
		school education (low - middle - high)(2)	1,266	1	0,261
		school education (low - middle - high)(3)	0,000	1	0,992
		have you ever worked in the health care-sector?(1)	1,407	1	0,236
		have you ever participated in research?(1)	0,051	1	0,821
		nationality (binary coding)(1)	1,404	1	0,236
		ever heard of biobanks before (0/1-coding)(1)	0,003	1	0,955
	Overall Statistics		31,071	16	0,013

Block 1: Method = Enter

Omnibus Tests of Model Coefficients

		Chi-square	df	Sig.
Step 1	Step	29,799	16	0,019
	Block	29,799	16	0,019
	Model	29,799	16	0,019

Model Summary

Step	-2 Log likelihood	Cox & Snell R Square	Nagelkerke R Square
1	139,890[a]	0,162	0,255

a. Estimation terminated at iteration number 5 because parameter estimates changed by less than ,001.

Hosmer and Lemeshow Test

Step	Chi-square	df	Sig.
1	5,030	8	0,754

Contingency Table for Hosmer and Lemeshow Test

		var_10dicho willingness to participate in biobank-research (yes - no - not sure) = 0 no (probably/certainly not)		var_10dicho willingness to participate in biobank-research (yes - no - not sure) = 1 yes (probably/certainly		Total
		Observed	Expected	Observed	Expected	
Step 1	1	10	10,549	7	6,451	17
	2	8	6,337	10	11,663	18
	3	4	4,612	13	12,388	17
	4	3	3,509	14	13,491	17
	5	4	2,653	13	14,347	17
	6	3	2,051	14	14,949	17
	7	1	1,597	16	15,403	17
	8	0	1,211	17	15,789	17
	9	0	0,885	17	16,115	17
	10	1	0,597	14	14,403	15

Classification Table[a]

Observed			Predicted var_10dicho willingness to participate in biobank-research (yes - no - not sure)		Percentage Correct
			0 no (probably/certainly not)	1 yes (probably/certainly	
Step 1	var_10dicho willingness to participate in biobank-research (yes - no - not sure)	0 no (probably/certainly not)	8	26	23,5
		1 yes (probably/certainly	5	130	96,3
	Overall Percentage				81,7

a. The cut value is ,500

Variables in the Equation

		B	S.E.	Wald	df	Sig.	Exp(B)
Step 1[a]	assessment of genetic research (approve - disapprove - not sure)			12,809	2	0,002	
	assessment of genetic research (approve - disapprove - not sure)(1)	-1,845	0,734	6,321	1	0,012	0,158
	assessment of genetic research (approve - disapprove - not sure)(2)	0,232	0,609	0,145	1	0,703	1,261
	age-groups (steps of 10 years)			7,533	6	0,274	
	age-groups (steps of 10 years)(1)	-1,009	0,832	1,471	1	0,225	0,364
	age-groups (steps of 10 years)(2)	0,447	0,855	0,274	1	0,601	1,564
	age-groups (steps of 10 years)(3)	0,145	0,774	0,035	1	0,851	1,156
	age-groups (steps of 10 years)(4)	-0,955	0,789	1,466	1	0,226	0,385
	age-groups (steps of 10 years)(5)	0,164	0,900	0,033	1	0,856	1,178
	age-groups (steps of 10 years)(6)	-1,173	0,922	1,617	1	0,203	0,309
	gender(1)	-0,880	0,492	3,189	1	0,074	0,415
	school education (low - middle - high)			0,459	3	0,928	
	school education (low - middle - high)(1)	0,204	0,886	0,053	1	0,818	1,226
	school education (low - middle - high)(2)	-0,150	0,825	0,033	1	0,856	0,861
	school education (low - middle - high)(3)	0,217	1,202	0,033	1	0,857	1,242
	have you ever worked in the health care-sector?(1)	-0,643	0,527	1,492	1	0,222	0,525
	have you ever participated in research?(1)	0,131	0,638	0,042	1	0,837	1,140
	nationality (binary coding)(1)	-0,417	0,745	0,313	1	0,576	0,659
	ever heard of biobanks before (0/1-coding)(1)	0,011	0,481	0,001	1	0,981	1,011
	Constant	2,647	1,069	6,134	1	0,013	14,114

a. Variable(s) entered on step 1: assessment of genetic research (approve - disapprove - not sure), age-groups (steps of 10 years), gender, school education (low - middle - high), have you ever worked in the health care-sector?, have you ever participated in research?, nationality (binary coding), ever heard of biobanks before (0/1-coding).

Logistic Regression; dependent variable: "hypothetical willingness to donate biomaterial and/or data"
Method: Forward Stepwise (Wald)

Notes

Output Created		l-2017 13:14:04
	N of Rows in Working Data File	204
Missing Value Handling	Definition of Missing	User-defined missing values are treated as missing
Syntax		LOGISTIC REGRESSION VARIABLES var_10dicho<br>/METHOD=FSTEP(WALD) var_3dicho age_gr Var_12 edu_grob health_job research_exp nat_dicho var_9adicho<br>/CONTRAST (var_3dicho)=Indicator<br>/CONTRAST (age_gr)=Indicator(1)<br>/CONTRAST (Var_12)=Indicator(1)<br>/CONTRAST (edu_grob)=Indicator(1)<br>/CONTRAST (health_job)=Indicator(1)<br>/CONTRAST (research_exp)=Indicator(1)<br>/CONTRAST (nat_dicho)=Indicator(1)<br>/CONTRAST (var_9adicho)=Indicator(1)<br>/PRINT=GOODFIT<br>/CRITERIA=PIN(0.05) POUT(0.10) ITERATE(20) CUT(0.5).

Case Processing Summary

Unweighted Cases[a]		N	Percent
Selected Cases	Included in Analysis	169	82,8
	Missing Cases	35	17,2
	Total	204	100,0
Unselected Cases		0	0,0
Total		204	100,0

a. If weight is in effect, see classification table for the total number of cases.

Dependent Variable Encoding

Original Value	Internal Value
0 no (probably/certainly not)	0
1 yes (probably/certainly	1

Categorical Variables Codings

		Frequency	Parameter coding (1)	(2)	(3)	(4)	(5)	(6)
age_gr age-groups (steps of 10 years)	1 18-29	27	0,000	0,000	0,000	0,000	0,000	0,000
	2 30-39	17	1,000	0,000	0,000	0,000	0,000	0,000
	3 40-49	25	0,000	1,000	0,000	0,000	0,000	0,000
	4 50-59	41	0,000	0,000	1,000	0,000	0,000	0,000
	5 60-69	20	0,000	0,000	0,000	1,000	0,000	0,000
	6 70-79	29	0,000	0,000	0,000	0,000	1,000	0,000
	7 80 oder älter	10	0,000	0,000	0,000	0,000	0,000	1,000
edu_grob school education (low - middle - high)	1 low (kein Abschluss/Hauptschule)	18	0,000	0,000	0,000			
	2 middle (Realschule)	41	1,000	0,000	0,000			
	3 high ((Fach)-Abitur)	100	0,000	1,000	0,000			
	8 other/still going to school	10	0,000	0,000	1,000			
var_3dicho assessment of genetic research (approve - disapprove - not sure)	0 disapprove (somewhat/definitely)	27	1,000	0,000				
	1 approve (somewhat/definitely)	111	0,000	1,000				
	3 not sure	31	0,000	0,000				
health_job have you ever worked in the health care-	0 no, never	132	0,000					
	1 yes, currently/before	37	1,000					
nat_dicho nationality (binary coding)	1 only German	157	0,000					
	2 German & other / only other	12	1,000					
Var_12 gender	1 male	73	0,000					
	2 female	96	1,000					
research_exp have you ever participated in research?	0 no	142	0,000					
	1 yes	27	1,000					
var_9adicho ever heard of biobanks before (0/1-coding)	0 no	115	0,000					
	1 yes	54	1,000					

Block 0: Beginning Block

Classification Table[a,b]

Observed			Predicted: var_10dicho willingness to participate in biobank-research (yes - no - not sure) 0 no (probably/certainly not)	1 yes (probably/certainly	Percentage Correct
Step 0	var_10dicho willingness to participate in biobank-research (yes - no - not sure)	0 no (probably/certainly not)	0	34	0,0
		1 yes (probably/certainly	0	135	100,0
	Overall Percentage				79,9

a. Constant is included in the model.

b. The cut value is ,500

Variables in the Equation

		B	S.E.	Wald	df	Sig.	Exp(B)
Step 0	Constant	1,379	0,192	51,642	1	0,000	3,971

Variables not in the Equation

			Score	df	Sig.
Step 0	Variables	assessment of genetic research (approve - disapprove - not sure)	15,754	2	0,000
		assessment of genetic research (approve - disapprove - not sure)(1)	15,710	1	0,000
		assessment of genetic research (approve - disapprove - not sure)(2)	6,548	1	0,011
		age-groups (steps of 10 years)	11,520	6	0,074
		age-groups (steps of 10 years)(1)	1,016	1	0,314
		age-groups (steps of 10 years)(2)	0,310	1	0,578
		age-groups (steps of 10 years)(3)	1,013	1	0,314
		age-groups (steps of 10 years)(4)	5,580	1	0,018
		age-groups (steps of 10 years)(5)	2,081	1	0,149
		age-groups (steps of 10 years)(6)	2,614	1	0,106
		gender(1)	3,296	1	0,069
		school education (low - middle - high)	1,418	3	0,701
		school education (low - middle - high)(1)	1,013	1	0,314
		school education (low - middle - high)(2)	1,266	1	0,261
		school education (low - middle - high)(3)	0,000	1	0,992
		have you ever worked in the health care-sector?(1)	1,407	1	0,236
		have you ever participated in research?(1)	0,051	1	0,821
		nationality (binary coding)(1)	1,404	1	0,236
		ever heard of biobanks before (0/1-coding)(1)	0,003	1	0,955
	Overall Statistics		31,071	16	0,013

Block 1: Method = Forward Stepwise (Wald)

Omnibus Tests of Model Coefficients

		Chi-square	df	Sig.
Step 1	Step	13,348	2	0,001
	Block	13,348	2	0,001
	Model	13,348	2	0,001
Step 2	Step	5,874	1	0,015
	Block	19,222	3	0,000
	Model	19,222	3	0,000

Model Summary

Step	-2 Log likelihood	Cox & Snell R Square	Nagelkerke R Square
1	156,341[a]	0,076	0,120
2	150,467[b]	0,108	0,170

a. Estimation terminated at iteration number 4 because parameter estimates changed by less [illegible]

b. Estimation terminated at iteration number 5 because parameter estimates changed by less [illegible]

Hosmer and Lemeshow Test

Step	Chi-square	df	Sig.
1	0,000	1	1,000
2	2,713	4	0,607

Contingency Table for Hosmer and Lemeshow Test

		[illegible] participate in biobank-research		[illegible] participate in biobank-research (yes [illegible]		
		Observed	Expected	Observed	Expected	Total
Step 1	1	13	13,000	14	14,000	27
	2	5	5,000	26	26,000	31
	3	16	16,000	95	95,000	111
Step 2	1	6	7,507	6	4,493	12
	2	7	5,493	8	9,507	15
	3	5	4,204	16	16,796	21
	4	13	12,289	50	50,711	63
	5	0	0,796	10	9,204	10
	6	3	3,711	45	44,289	48

Classification Table[a]

Observed			Predicted: var_10dicho willingness to participate in biobank-research (yes - no - not sure)		Percentage Correct
			0 no (probably/certainly not)	1 yes (probably/certainly	
Step 1	var_10dicho willingness to participate in biobank-research (yes - no - not sure)	0 no (probably/certainly not)	0	34	0,0
		1 yes (probably/certainly	0	135	100,0
	Overall Percentage				79,9
Step 2	var_10dicho willingness to participate in biobank-research (yes - no - not sure)	0 no (probably/certainly not)	6	28	17,6
		1 yes (probably/certainly	6	129	95,6
	Overall Percentage				79,9

a. The cut value is ,500

Variables in the Equation

		B	S.E.	Wald	df	Sig.	Exp(B)
Step 1[a]	assessment of genetic research (approve - disapprove - not sure)			13,810	2	0,001	
	assessment of genetic research (approve - disapprove - not	-1,575	0,622	6,409	1	0,011	0,207
	assessment of genetic research (approve - disapprove - not	0,133	0,558	0,056	1	0,812	1,142
	Constant	1,649	0,488	11,398	1	0,001	5,200
Step 2[b]	assessment of genetic research (approve - disapprove - not sure)			15,598	2	0,000	
	assessment of genetic research (approve - disapprove - not	-1,899	0,658	8,327	1	0,004	0,150
	assessment of genetic research (approve - disapprove - not	0,032	0,566	0,003	1	0,955	1,033
	gender(1)	-1,062	0,461	5,302	1	0,021	0,346
	Constant	2,447	0,623	15,452	1	0,000	11,557

a. Variable(s) entered on step 1: assessment of genetic research (approve - disapprove - not sure).

b. Variable(s) entered on step 2: gender.

Variables not in the Equation

			Score	df	Sig.
Step 1	Variables	age-groups (steps of 10 years)	10,144	6	0,119
		age-groups (steps of 10 years)(1)	2,308	1	0,129
		age-groups (steps of 10 years)(2)	1,338	1	0,247
		age-groups (steps of 10 years)(3)	1,233	1	0,267
		age-groups (steps of 10 years)(4)	3,359	1	0,067
		age-groups (steps of 10 years)(5)	0,830	1	0,362
		age-groups (steps of 10 years)(6)	2,417	1	0,120
		gender(1)	5,572	1	0,018
		school education (low - middle - high)	1,287	3	0,732
		school education (low - middle - high)(1)	0,491	1	0,483
		school education (low - middle - high)(2)	1,194	1	0,274
		school education (low - middle - high)(3)	0,416	1	0,519
		have you ever worked in the health care-sector?(1)	2,530	1	0,112
		have you ever participated in research?(1)	0,000	1	0,995
		nationality (binary coding)(1)	0,480	1	0,488
		ever heard of biobanks before (0/1-coding)(1)	0,027	1	0,869
	Overall Statistics		16,307	14	0,295
Step 2	Variables	age-groups (steps of 10 years)	8,571	6	0,199
		age-groups (steps of 10 years)(1)	2,222	1	0,136

		age-groups (steps of 10 years)(2)	1,149	1	0,284
		age-groups (steps of 10 years)(3)	1,050	1	0,306
		age-groups (steps of 10 years)(4)	2,476	1	0,116
		age-groups (steps of 10 years)(5)	0,860	1	0,354
		age-groups (steps of 10 years)(6)	1,938	1	0,164
		school education (low - middle - high)	1,306	3	0,728
		school education (low - middle - high)(1)	0,339	1	0,561
		school education (low - middle - high)(2)	1,050	1	0,306
		school education (low - middle - high)(3)	0,676	1	0,411
		have you ever worked in the health care-sector?(1)	1,245	1	0,265
		have you ever participated in research?(1)	0,083	1	0,773
		nationality (binary coding)(1)	0,667	1	0,414
		ever heard of biobanks before (0/1-coding)(1)	0,032	1	0,857
	Overall Statistics		10,945	13	0,615
